# High-throughput spatiotemporal monitoring of single-cell secretions via plasmonic microwell arrays

**DOI:** 10.1038/s41551-023-01017-1

**Published:** 2023-04-03

**Authors:** Saeid Ansaryan, Yen-Cheng Liu, Xiaokang Li, Augoustina Maria Economou, Christiane Sigrid Eberhardt, Camilla Jandus, Hatice Altug

**Affiliations:** 1 Institute of Bioengineering, École Polytechnique Fédérale de Lausanne (EPFL), Lausanne, Switzerland; 2 Department of Oncology, Centre Hospitalier Universitaire Vaudois (CHUV), Lausanne, Switzerland; 3 Ludwig Institute for Cancer Research, Lausanne Branch, Agora Center, Lausanne, Switzerland; 4 Center for Vaccinology, University Hospitals Geneva and University of Geneva, Geneva, Switzerland; 5 Division of General Pediatrics, Department of Woman, Child and Adolescent Medicine, Faculty of Medicine, University of Geneva, Geneva, Switzerland; 6 Department of Pathology and Immunology, University of Geneva, Geneva, Switzerland

**Keywords:** Proteomics, Optical imaging

## Abstract

Methods for the analysis of cell secretions at the single-cell level only provide semiquantitative endpoint readouts. Here we describe a microwell array for the real-time spatiotemporal monitoring of extracellular secretions from hundreds of single cells in parallel. The microwell array incorporates a gold substrate with arrays of nanometric holes functionalized with receptors for a specific analyte, and is illuminated with light spectrally overlapping with the device’s spectrum of extraordinary optical transmission. Spectral shifts in surface plasmon resonance resulting from analyte–receptor bindings around a secreting cell are recorded by a camera as variations in the intensity of the transmitted light while machine-learning-assisted cell tracking eliminates the influence of cell movements. We used the microwell array to characterize the antibody-secretion profiles of hybridoma cells and of a rare subset of antibody-secreting cells sorted from human donor peripheral blood mononuclear cells. High-throughput measurements of spatiotemporal secretory profiles at the single-cell level will aid the study of the physiological mechanisms governing protein secretion.

## Abstract (web summary)

A plasmonic microwell array incorporating a gold substrate with arrays of receptor-coated nanometric holes enables the imaging-based spatiotemporal monitoring of secreted proteins from hundreds of single cells at sub-minute resolution.

## Main

Cellular communications mediated by protein secretion are responsible for a plethora of important physiological functions, such as growth and proliferation^1^, metabolic regulation^2^, immune response^3^ and even different cell-death modalities^4–6^. For example, in the case of the immune system, the activation efficiency and specificity of the response can be controlled by the temporal and spatial characteristics of the released soluble factors, such as duration and frequency in time, as well as direction and distribution in extracellular space^7–10^. Another important consideration is the inherent heterogeneity of the protein secretions (collectively referred to as ‘secretome’^11^) on a cell-to-cell basis in terms of their spatial and temporal distribution as well as function. Secretomic heterogeneity has been observed both within phenotypically homogeneous cell populations^12,13^ and between distinct populations of healthy and pathological cells^14,15^. Consequently, understanding the inner workings of cellular secretion is decisive in investigating processes in cell biology^16^, in exploring the evolution of dynamic diseases such as cancer^17,18^, and in developing new pharmacological therapies^19^.

However, the comprehensive study of the cellular secretome cannot be achieved without quantitative and real-time analysis at the single-cell level. Such methods should entail a high spatial resolution to differentiate individual cells and spatially visualize the distribution of the secreted proteins, and should also have a high temporal resolution to identify rapid or minute alterations in secretion^20^. Furthermore, a large number of cells should be investigated simultaneously for sufficient statistical significance while discriminating stochastic biological noise^21^. Conventional methods such as enzyme-linked immunospot (ELISpot) and intracellular cytokine staining (ICS) can detect protein production from single cells with high throughput. However, they provide endpoint results that hinder them from extracting kinetic information owing to the lack of sufficient temporal resolution^22^. ICS fails to distinguish between produced and actually secreted proteins to the external medium and damages cell viability, precluding the use of cells for downstream analysis^23^. ELISpot can capture secreted factors from live cells in the extracellular space, but its spatial resolution is insufficient to resolve secretion direction and distribution^24^. Continuous advances in microfluidics made in the past decade have empowered the capture, compartmentalization and processing of single cells to profile their secretory properties^22,25–27^. Relevant high-throughput systems include microengraving^28,29^, droplet-based screening assays^30,31^ and chamber-based barcoding chips^12^, which minimize the assay volume required to obtain a detectable concentration of the target molecules. However, these systems usually rely on fluorescent label-based detection methods, which inherently reduce the spatial and temporal resolution owing to multistep labelling and washing processes in the formation of antigen–antibody immunocomplexes.

Label-free optical detection methods hold great promise for biosensing and single-cell studies^32–34^. In particular, the surface plasmon resonance (SPR) principle has been widely adopted and commercialized for the real-time quantification of biomolecule interactions both in biosensing and bioimaging configurations^32,35^. However, conventional SPR sensors typically require a precisely aligned prism-coupling configuration for the excitation of plasmons, hindering system integration due to the bulky optics^35^. For the implementation of SPR imaging, oblique angle light incidence to prism compromises the quality of the obtained images and the spatial resolution due to optical aberrations^33,36^. Recent advances in nanotechnology have facilitated the miniaturization of nanophotonic detection systems^37,38^, providing outstanding sensitivity by supporting strong light–matter interactions^39,40^. However, they have been used so far with a limited capacity for single-cell secretion analysis. The ultrasensitive and highly specific detection of cytokine secretion at the single-cell level was shown by measuring resonance-wavelength shifts with high-resolution spectroscopy. Despite its performance, the small field of view due to the narrow slit in spectroscopy and the low throughput hamper the extraction of the spatial distribution of secretions around single cells over extended areas. Alternatively, photonic-crystal resonant imaging was implemented to map the secretion of signalling proteins from single cells and to model cell-adsorption kinetics^41^. Nevertheless, the amount of secretion was not quantified, the duration of the experiment was only up to 2 h, and the throughput improvement was low, with a few tens of cells.

In this work, we introduce a label-free nanoplasmonic imaging system that enables the spatiotemporal mapping of single-cell secretions in a microwell-array format. The biosensor comprises gold nanohole arrays that support a highly sensitive extraordinary optical transmission (EOT) spectrum for detection and a two-dimensional (2D) array of polymeric microwells that allows the deterministic loading of many individual cells. To achieve high-throughput analysis, we directly measure the intensity variations resulting from the changes in the EOT spectrum upon the binding of secreted analytes on the sensor surface as a function of time over a large field of view (FOV) using a scientific complementary metal-oxide-semiconductor (sCMOS) camera and without requiring spectroscopy. This label-free readout method, which generates time-resolved and large-area intensity images from millions of pixels, is augmented with unsupervised image-processing techniques and a machine-learning algorithm to construct ‘spatiotemporal secretion maps’ in four dimensions (*x*, *y*, intensity, time) while simultaneously tracking the motility and morphology of each individual cell. Our computational pipeline allows for accurate visualization, quantification and prolonged analysis of secreted products in the extracellular space for hundreds of individual cells.

We first validated the system by tracking the immunoglobulin G (IgG) secretion of genetically identical engineered hybridoma cells (as a well-characterized model cell line) at the single-cell level for the remarkably long observation times of over 12 h, and with a temporal resolution of minutes. We were able to extract the spatial distribution of the secreted products around the cells over the surface and observed various secretory patterns. Additionally, we investigated how protein transport is affected during cell division by mapping the spatiotemporal changes in secretion for mitotic cells. Next, we analysed the spatiotemporal distribution of the total content release of cancer cells following different cell-death pathways and obtained distinct signatures, providing insights into their underlying mechanisms. Finally, we demonstrated the broad applicability of the system for the analysis of primary human samples by studying the secretion of peripheral blood mononuclear cells (PBMCs). The capability to extract the temporal and spatial profile of the released factors through spatiotemporal maps could facilitate the study of cell-signalling mechanisms. The system is compatible with both adherent and suspended cells, and can be adapted to detect different types of secreted molecule, including (but not limited to) exosomes, cytokines and antibodies. Therefore, it represents a powerful and versatile analytical tool for supporting the discovery of previously uncharacterized or unknown cell-to-cell secretory variations in both homogeneous and heterogeneous populations.

## Results and Discussion

### Working principle of the plasmonic microwell array system

**Fig. 1:**
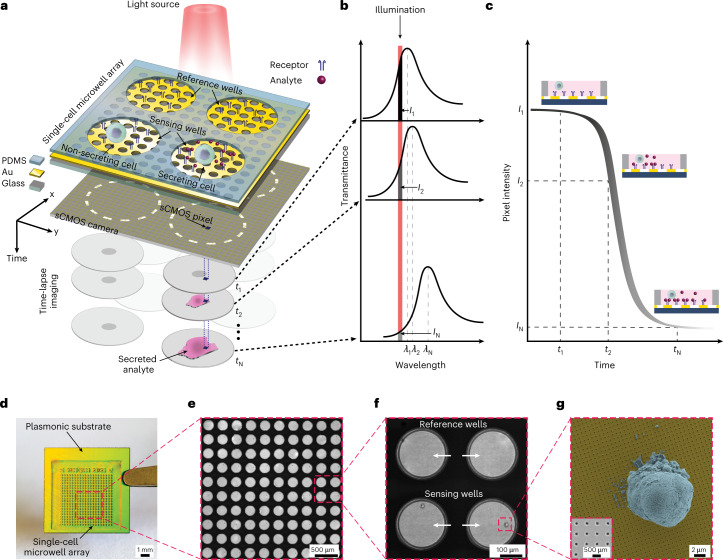
Plasmonic single-cell microwell array system. **a**,**b**, Schematic of the working principle of the plasmonic single-cell microwell array showing all four main elements: LED, PDMS micromesh, gold nanohole array substrate and sCMOS camera. Gold nanohole arrays are functionalized with desired receptors against a specific analyte secreted by the cells into the extracellular space. By illuminating the gold nanohole array substrate with the LED, for a secreting cell, the interactions between the analytes and receptors alter the refractive index on the close vicinity of the surface and consequently, the resonance peak redshifts. The sCMOS camera collects time-lapse images with a sub-minute temporal resolution to translate the spectral shifts into the intensity changes in the camera’s pixels over time. **c**, A 1D binding event sensogram at each camera pixel is obtained by measuring the intensity changes in real time. High-efficiency light transmission through the patterned gold surface provides high-contrast optical images enabling simultaneous analysis of secretion and morphology of the cell. **d**, Photograph of the integrated plasmonic gold nanohole array substrate with the PDMS micromesh featuring 20 × 20 arrays of microwells. **e**, Image of the plasmonic single-cell microwell array after deterministic loading of the individual cells using the piezoelectric liquid dispenser. **f**, Representative image of the single-cell microwell array showing the sensing wells containing individual cells and empty reference wells used for noise reduction. **g**, SEM image of a hybridoma cell on the nanohole array substrate. Nanoholes are patterned throughout the substrate, allowing secretion monitoring regardless of cell location. The inset shows nanoholes with a diameter of 200 nm and periodicity of 600 nm.

Figure 1a–c schematically illustrates the working principle of the system. The plasmonic single-cell microwell array consists of four main parts: a plasmonic gold nanohole array substrate, a polydimethylsiloxane (PDMS) micromesh for single-cell compartmentalization, a light-emitting diode (LED) as the illumination source and an sCMOS camera (Fig. 1a). We exploit plasmon excitation at a normal incidence on nanohole arrays through a grating coupling mechanism to implement sensing with a robust and easy-to-maintain collinear optical scheme. This enables us to leverage a conventional inverted microscope and double-purpose its existing parts (LED, camera, objective) for both optical and plasmonic imaging. By illuminating the nanohole arrays with a narrowband light source that spectrally overlaps with part of the EOT spectrum, the resonance shifts resulting from analyte–receptor bindings around a secreting cell are manifested as spatially resolved intensity variations of the transmitted light and recorded as images by the camera (Fig. 1a,b). The sCMOS camera along with a medium power magnification objective enables us to have a large FOV of 1.1 mm × 0.65 mm while collecting the intensity variation of every single pixel. Furthermore, the fast image acquisition and stage movement, which last for tens of milliseconds, allow us to implement automated sample scanning and acquire images from hundreds of positions with sub-minute temporal resolution (see Supplementary Discussion 1 for details on temporal resolution). By collecting these time-lapse plasmonic intensity images at each position (Fig. 1a), we process the corresponding intensity changes over time (Fig. 1b) and extract the binding event sensogram (Fig. 1c) over the entire chip surface. Remarkably, we observe that the efficiency of light transmission (within a narrow band of the EOT spectrum) through an optically thick gold film (~120 nm) is strong enough to perform optical imaging of the cells when the film is perforated with nanohole arrays. In contrast, a uniform gold film without nanostructures acts as a mirror and allows almost no light transmission. We used this feature to concurrently monitor the morphological changes of the cells while detecting their secretion profiles through plasmonics.

For single-cell manipulation, we incorporated an easy-to-handle PDMS micromesh to form arrays of open-top microwells on the plasmonic substrate and employed compartmentalization of a large number of single cells in an array format. Figure 1d shows the integrated plasmonic single-cell microwell array with 20 × 20 microwells of 200 μm diameter on the gold nanohole array substrate.

We accomplish deterministic single-cell seeding using a piezoelectric liquid dispenser (see Supplementary Discussion 2 and Methods for more details), bypassing random cell seeding on the basis of Poisson loading. The dispenser is coupled with advanced image processing for real-time and high-accuracy single-cell isolation and loading. Figure 1e shows the microwell array after seeding the single cells. Figure 1f displays the zoomed view of the microwell array, highlighting that for each well containing a single cell, there is a corresponding reference well (50% reference occupancy) for background correction. It should be noted that the design of the microwell array (for example, substrate size, well diameter, well periodicity and reference occupancy) and the image acquisition settings (for example, exposure/stabilization time, FOV and stage control) affect the device characteristics including throughput and temporal resolution. Depending on cell size, required area for the distribution of the secreted products in the extracellular space and cell movement, such characteristics can be optimized to address the need of the biological question (Supplementary Discussion 1).

Figure 1g shows scanning electron microscopy (SEM) of a hybridoma cell on the gold nanohole array substrate. Nanohole arrays with a diameter of 200 nm and periodicity of 600 nm are fabricated on a 4-inch wafer using a deep ultraviolet (DUV) photolithography process, and the wafer is diced to produce 1 cm × 1 cm plasmonic substrates. This configuration featuring nanoholes over the entire substrate effectively makes every point on the surface a sensing element and gives the flexibility to visualize the spatial distribution of extracellular secretion and morphological behaviours of the individual cells irrespective of their positions. We evaluated the optical uniformity of the nanoholes by characterizing the spectral peak position and full-width-half-maximum (FWHM) of the EOT resonances across the entire wafer. Our results show a high-level uniformity and robustness for the fabrication, with the resonance peak position (at 860.7 nm in water) and FWHM (22.6 nm) varying less than 2.1 nm and 0.8 nm over different wafers, respectively (Supplementary Fig. 3c).

### 4D spatiotemporal secretion analysis

We first apply the system to engineered hybridoma cells (anti-mouse CD45.1) as a well-characterized model cell line to demonstrate a quantitative analysis of secretion kinetics with spatial and temporal information. Hybridomas are vastly used to produce monoclonal IgG antibodies, with a wide range of diagnostic and therapeutic applications^42,43^.

**Fig. 2:**
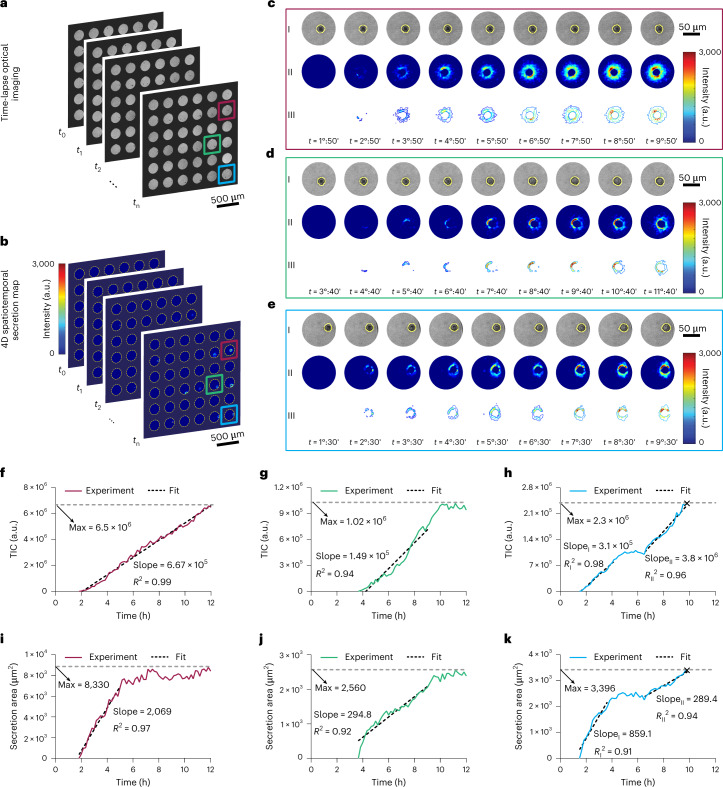
4D spatiotemporal secretion map allows for the identification of distinct secretion profiles in hybridoma cells. **a**, Time-lapse optical images of the single-cell microwell array are used to track the cells’ positions and monitor their morphological changes using a machine-learning random forest algorithm. **b**, 4D spatiotemporal secretion maps of the cells in the microwell array over time. The map shows variations in the intensity of the camera pixels due to the spectral redshifts originating from IgGs binding on the sensor surface. **c**–**e**, Three representative secretion profiles for the hybridoma cells along with their corresponding optical images show a gradual increase in both intensity and area covered by the secretion over selected time points. I, bright-field optical images; II, spatiotemporal secretion maps; III, secretion contour plots. In the optical images, the cell boundary is displayed by the yellow lines around the cell, using the cell tracking algorithm in the machine-learning protocol. **f**–**h**, TIC curves indicating the amount of IgGs secreted over time for the cells shown in **c**,**d** and **e**, respectively. Linear curve fitting is used to extract the secretion rate (the slope), and the maximum amounts of secretions (Max) are denoted by the grey dashed lines. **f** shows a linear increase in secretion during 12 h (type I), while the secretion in **g** reaches a plateau after some hours and forms a half sigmoid (type II). **h** is a combination of these two secretion profiles as it shows another rise after the plateau (type III). **i**–**k**, Secretion area curves presenting the area occupied by the adsorbed IgGs on the sensor surface over time for the cells shown in **c**,**d** and **e**, respectively. The adsorption rates (indicated by the slope) and the maximum secretion areas (given by Max) are calculated in a similar way as the TIC curves. The × sign on the curves refers to the onset of apoptosis.

Figure 2a shows optical images of a subset of the single-cell microwell array after loading the hybridomas. We performed time-lapse optical imaging of the microwell array with a 10 min time interval for 12 h. Using a machine-learning random forest algorithm, the morphology and migratory behaviour of the cells were monitored and their boundaries in each time frame were extracted and used for subsequent image processing. To specifically detect the IgGs secreted by the cells, we immobilized protein A/G on the surface, which is a common choice for antibody purification^44^ providing cumulative properties of both protein A and G to selectively capture the total IgGs. The functionalization process and its uniformity are discussed in Methods and in Supplementary Discussions 3 and 4.

The system can image the entire microwell and the area surrounding each cell as a function of time with high temporal resolution. We took advantage of this feature to introduce a four-dimensional (4D) spatiotemporal secretion map (*x*, *y*, intensity, time) for single cells (Fig. 2b). This was generated using machine-learning-assisted image processing to show intensity changes for the sCMOS pixels resulting from the spectral shifts due to the binding interactions on the plasmonic substrate. It also enabled exclusion of cells from the 4D secretion map to only exhibit variations in the intensity of the transmitted light due to the binding of secreted IgGs to the surface without the interference of the cell mass. Zoomed-in views of the 4D secretion maps along with optical images of three representative single hybridomas at selected time points are displayed in Fig. 2c–e (see Supplementary Videos 1–6).

Secretion maps (the second rows in Fig. 2c,d) show the distribution pattern of the produced IgGs in the extracellular space over the surface as a function of time. To facilitate comparison of secretory patterns among different cells, we also included contour plots which are constructed from the secretion maps by extracting the distribution of the secreted products at discrete levels of intensity (the third rows in Fig. 2c,d). In Fig. 2d, secretion followed a spatial pattern resembling a C shape for almost 6 h and developed a symmetric O shape at the end of the observation period. On the other hand, a relatively symmetric secretion was seen for the cells shown in Fig. 2c,e. Using the cell tracking algorithm, we analysed the migration trajectory for the three representative cells in Fig. 2c–e (Supplementary Figs. 6a–c). Since the mean squared displacements were quite negligible (0.95, 1.85 and 1.12 μm^2^ for Fig. 2c–e, respectively), cell motility could not have altered the analysis of the secretion maps for the tested hybridoma cells. For other types of cell that are highly motile, the compatibility of our plasmonic substrates with microfluidic integration provides an advantage for incorporation of a cell entrapment unit to partially immobilize the cells during spatiotemporal secretion monitoring^45^. The capability of the system to visualize secretion distribution outside of the cells could benefit the study of cell signalling in which communication can be controlled by the spatial profile of the released factors. For example, a multidirectional secretion by the immune cells promotes inflammation in the immune responses, whereas a directional secretion of cytokines toward their target cells facilitates specific communications^7,10^.

To extract a wide range of kinetic information and secretion dynamics, we processed the 4D secretion maps in two ways. First, we calculated the total intensity change (TIC) in each well by summing the absolute intensity changes of all the pixels at each time frame (Fig. 2f–h). TIC curves are associated with the amount of IgGs secreted by the cells as a function of time. Second, we calculated the secretion area as the number of non-zero pixels in each secretion map frame multiplied by the physical area of each pixel (Fig. 2i–k). Secretion area curves provide information about the spatial extent of the secreted IgGs adsorbed to the sensor surface around a given cell as a function of time. The *x* axis of the TIC and secretion area curves is the running time of the experiment, which lasted for 12 h, and the starting point of each curve is the onset of secretion.

In Fig. 2f–h, the secretion rates of the single cells were extracted by fitting linear equations to the linear parts of the curves and calculating the slope of the fits (see Methods). The experimental maximum TIC values related to the maximum amount of secretion for the cells are indicated by the grey dashed lines. TIC curves are used to define three types of secretion profile: linear, half sigmoid and combined. The linear profile refers to the cells whose secretion curves follow a linear pattern (type I) (Fig. 2f). The half sigmoid one refers to the cells whose secretion curves eventually reach a plateau after experiencing the first increase (type II) (Fig. 2g). Some of the secreting cells exhibited a combination of these two profiles by showing a further rise in the secretion curves after the plateau time (type III) (Fig. 2h). Similar to the TIC curves, from the secretion area curves (Fig. 2i–k) we also extracted the adsorption rates of the IgGs to the substrate using linear curve fitting and calculated the slopes for the linear part of the curves. The experimental maximum secretion area values are also indicated by the grey dashed lines.

### Statistical analysis of secretion heterogeneity

**Fig. 3:**
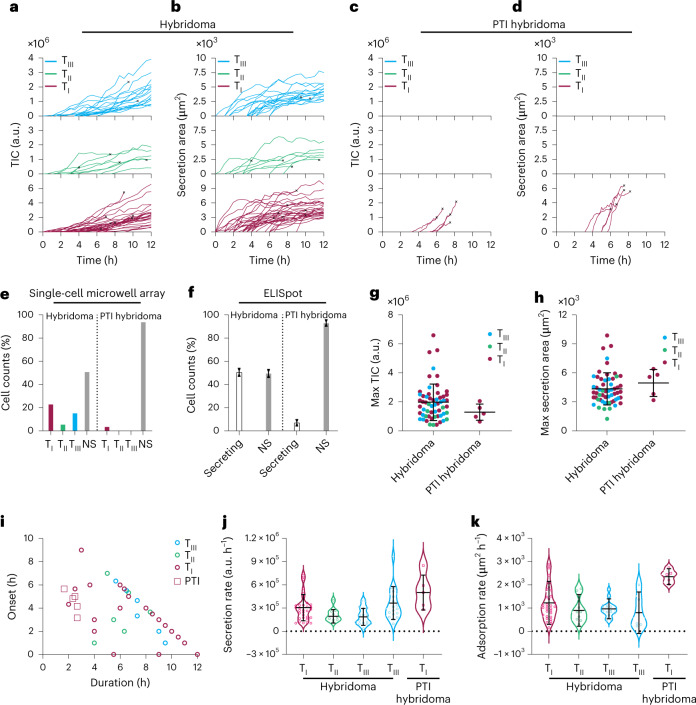
Statistical secretion analysis for a population of hybridoma cells. **a**,**b**, TIC (**a**) and secretion area curves (**b**) for untreated hybridoma cells showing the presence of all the three types of secretion. **c**,**d**, TIC (**c**) and secretion area curves (**d**) for PTI hybridomas with only a few secreting cells from type I. Each curve represents a single cell, and ‘×’ shows the apoptosis onsets. **e**, Comparison of secretion percentage for the three types of cell behaviour in untreated and PTI hybridomas. **f**, ELISpot results for both untreated and PTI cells, which agree with the total secretion percentage obtained by the plasmonic single-cell microwell array. Error bars represent s.d. between two replicates for each column as shown in Supplementary Fig. 8. **g**, A 1D scatterplot comparing the maximum TIC for both populations, illustrating a wider distribution (see Supplementary Table 1 for details) in the amount of secreted IgGs for the untreated cells. **h**, Maximum secretion areas for both populations emphasizing higher heterogeneity for the untreated one with a slightly lower average value (see Supplementary Table 1 for details). **i**, A 2D scatterplot showing the onset of secretion vs duration. It reveals that PTI cells secreted for shorter times and mainly within the first hours of the experiment before the secretory pathway got blocked completely. **j**,**k**, Secretion and adsorption rates of IgGs for the three types of cell behaviour in both populations. The large s.d.s with similar mean values (see Supplementary Table 1 for details) for the different secretion types in untreated hybridomas indicate the diversity of the cell behaviours, which can only be uncovered by monitoring the cells at single-cell resolution and extracting their kinetics. In total, 160 and 168 single-cell measurements were performed for the untreated and PTI-hybridoma populations, respectively. The error bars in **g**, **h**, **j** and **k** represent the mean ± s.d. of secreting cells from these populations, with *n* = 58 (32, 9 and 17 in the T1, T2 and T3 groups, respectively) for untreated hybridoma cells and *n* = 5 (only in the T1 group) for PTI-hybridoma cells.

To investigate the secretory behaviour of a large number of cells, we analysed a population of 160 single clonal hybridomas. As a control, we monitored the secretion of protein transport inhibited (PTI) hybridoma cells, which are treated with brefeldin A and monensin to prevent protein transport into the extracellular space. The results of both sets of experiments are presented in Fig. 3. Figure 3a,b show the TIC and secretion area curves for untreated secreting hybridomas with respect to their secretion type, as detailed previously. Figure 3c,d show the respective curves for the population of PTI hybridoma cells. Only a few PTI hybridomas exceptionally secreted IgGs by following a type I secretory behaviour. The substantial decrease in the number of secreting cells after receiving the inhibitory treatment indicates that the observed signals rely on the classical secretory pathway. The total number of secreting cells from the test and control cell populations is summarized in Fig. 3e, where linear secretion (type I) dominates in both populations. To evaluate the specificity of the system, we included two control experiments: hybridoma cells in the absence of protein A/G on the surface and human K562 lymphoblasts, secreting a wide range of other biomolecules (Supplementary Fig. 7a,b), on the protein A/G functionalized surface. As shown in Supplementary Fig. 7c,d, no notable increase in the TIC and secretion area curves is observed for these two controls (see Supplementary Discussion 5 and Methods for more details).

To validate the results obtained by the system, ELISpot assays were used to compare the percentage of secreting and non-secreting (NS) cells for both populations (Fig. 3f and Supplementary Fig. 8). We confirmed that 50.5% and 7% of the hybridoma and PTI hybridoma cells, respectively, secreted IgGs, which is consistent with the results obtained by the plasmonic single-cell microwell array. Figure 3e illustrates the advantages of the single-cell-analysis system for discerning the secretion heterogeneity in both populations (24.2% type I, 6.8% type II and 16.6% type III for the untreated hybridomas and 4.9% type I for the PTI ones).

To further compare the secretion profiles between the untreated and PTI hybridoma cells, the maximum experimental TIC and secretion area values for all the secreting cells in both populations are shown in one-dimensional (1D) scatterplots in Fig. 3g,h. The large standard deviation of the parameters for the untreated hybridomas demonstrated the heterogeneity of secretion in terms of maximum secretion and areal coverage (see Supplementary Table 1). As expected, the average maximum TIC of untreated hybridomas was higher than that of PTI cells, given the secretory pathway blockade in the latter. Notably, the secretions of PTI hybridomas covered more area on average than those of the untreated ones. We illustrated the capability for simultaneous monitoring of secretory and morphological changes by investigating the correlation between cell size and the obtained maximum values for TIC and secretion area. Since the cell morphology changed over time, we used the cell size at the onset of secretions to standardize analysis for all the secreting cells. Supplementary Fig. 9 indicates that no strong correlation was observed for this specific cell line. This capability could be useful for studying morphology-induced cell secretion variations, for instance, in mesenchymal and endothelial cells where changes in cell shape can regulate cytokine production^46,47^.

We then evaluated the link between the onset and duration of secretion (see Methods) for both untreated and PTI cell populations (Fig. 3i). PTI hybridomas secreted for a shorter duration during the first hours of the experiments before the inhibitory treatment reached its full efficacy. On the other hand, secretion in untreated hybridomas could last for up to 12 h. Here, it should be mentioned that we chose to capture the images every 10 min due to the long secretion duration of these cells. This microwell array configuration can achieve about a 1 min temporal resolution for screening 400 wells at 50% reference occupancy (Supplementary Fig. 1c). Due to the inherent trade-off between temporal resolution and throughput in microwell arrays, the design can be optimized further to improve such aspects. One example of secretion monitoring from an independent experiment with a denser microwell array (100 μm well diameter and 6.7% reference occupancy) is presented in Supplementary Fig. 10 (see Supplementary Videos 7 and 8), where the images were captured every 2 min and performance similar to that of the current design was achieved.

The distribution of the secretion rates for both populations is shown in Fig. 3j. As type III secretion has two linear parts, we reported separate secretion rates for each one. The PTI cells showed a higher mean value than the untreated cells. Similarly, the distribution of adsorption rates (Fig. 3k) showed higher values for the PTI cells compared with the untreated ones. An important observation to be made from Fig. 3j,k is that the large variations among the three types of secretions (Supplementary Table 1) hint at the heterogeneity of secretion in the hybridoma population. An ensemble averaging technique would only report the average secretion and adsorption rates that are approximately the same for all the three types, thereby leading to the false assumption that all hybridomas secrete in the same manner. The system demonstrates that population averages cannot capture the population states accurately and, in fact, obscure the true diversity of biological mechanisms such as secretion patterns among cells.

### IgG secretion and cell division

To show the applicability of the system for fundamental studies on cell biology, we studied single-cell secretion profiles during cell division when the secretory pathway undergoes several functional changes^48,49^. To understand how the secretory behaviour of a mother cell is inherited by the daughters and how protein secretion depends on the cell cycle, several studies have attempted to map the secretion of mother and daughter cells during division^50–53^. However, such studies analyse the secretion at a specific phase of division by inducing cell cycle arrest, which could disrupt cell function and alter secretory behaviour^54^. Additionally, they have been performed with bulk cell precision that can mask both the relevant variations in secretion and functionally rare events.

**Fig. 4:**
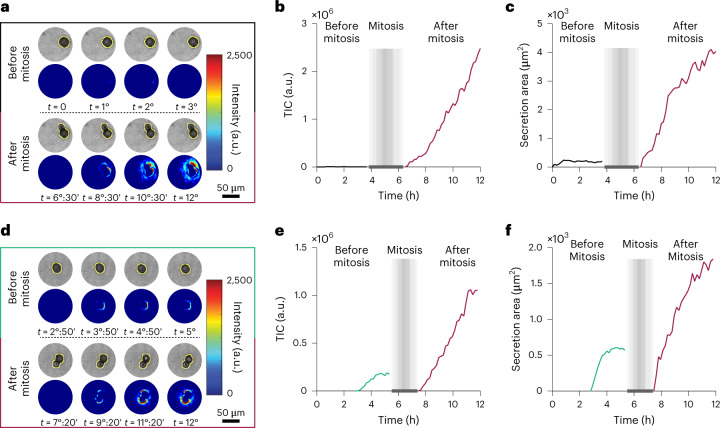
Spatiotemporal monitoring of IgG secretion in mother and daughter cells. **a**, 4D spatiotemporal secretion map for a hybridoma cell before and after division along with the corresponding optical images at selected time points that indicate secretion only for the daughter cells. The yellow lines around the cells define the boundary of the cells extracted by machine-learning algorithms. **b**, TIC curves that show type I cell behaviour for the daughter cells. **c**, Secretion area curves for the cells after mitosis. **d**, 4D spatiotemporal secretion map for a hybridoma cell before and after division with obvious secretion in both phases. **e**, TIC curves illustrating type II behaviour for the mother cell and type I behaviour for the daughter cells. **f**, Secretion area curves before and after mitosis. The grey boxes represent the time of mitosis and temporary changes in intensity due to cell movements before the cells settled down in their new positions.

We used the system to monitor the secretion dynamics of mother and daughter cells, owing to its ability to perform long-term and uninterrupted analysis of secretory and morphological behaviours. We revealed two distinct secretion profiles before and after cell division. Figure 4a shows the 4D secretion map for a hybridoma cell at selected time points in which the mother cell did not secrete before mitosis (Supplementary Videos 9 and 10) while both daughters started secreting IgGs right after mitosis (Supplementary Videos 11 and 12). Figure 4b presents the TIC curves for the mother and daughter cells, illustrating type I secretion after mitosis with 452,768 a.u. h^−1^ secretion rate. Figure 4c shows the secretion area curves with 754.2 μm^2^ h^−1^ adsorption rate of IgGs for the daughters. The cell tracking algorithm in our image-processing pipeline allows precise detection of the cell borders during mitosis and their exclusion from the subsequent analyses to eliminate temporary changes in the local refractive index and consequently, the intensity due to cell movement (shown as grey boxes in the curves).

In contrast, the 4D secretion map in Fig. 4d indicates secretion for both the mother (Supplementary Videos 13 and 14) and daughter cells (Supplementary Videos 15 and 16). According to the TIC curves (Fig. 4e), the mother cell underwent type II secretion (secretion rate of 112,209 a.u. h^−1^), while the daughters experienced type I secretion (at 270,405 a.u. h^−1^ secretion rate). Importantly, the plateau reached by the mother cell before mitosis is consistent with the downregulation of protein transport during mitosis^48^. Secretion area curves for both mother and daughters are shown in Fig. 4f, revealing adsorption rates of 351.2 μm^2^ h^−1^ and 392.6 μm^2^ h^−1^, respectively. As expected, the secretion and adsorption rates as well as the maximum TICs (266,655 a.u. for the mother and 1,061,615 a.u. for the daughters) and secretion areas (739.7 μm^2^ for the mother and 1,842.8 μm^2^ for the daughters) were higher for the two daughter cells than for the mother cell.

### Visualization of content release during cell death

Cell death, a critical stage in cells’ lifespan, results in the release of biomolecules that significantly influence the cellular surroundings. The released contents differ by modalities of death and have been shown to provoke distinct immune responses^6^. For instance, it is generally accepted that cells undergoing necroptosis, a caspase-independent programmed cell death, are more immunogenic than apoptotic ones due to the release of proinflammatory factors into the extracellular space generating so-called ‘find-me’ and/or ‘eat-me’ signals for the immune cells^6,55^. In this regard, by monitoring the released contents during cell death with a high spatiotemporal resolution, the system can provide insights into the underlying mechanisms of cell death. Especially, the label-free feature of the system enables capture of the signature of the total content release during cell death instead of monitoring one specific target with fluorescence imaging. To show this capability, we examined the behaviour of K562 cells, a myelogenous leukaemia cell line, during apoptosis and necroptosis, which are two important types of programmed cell death.

**Fig. 5:**
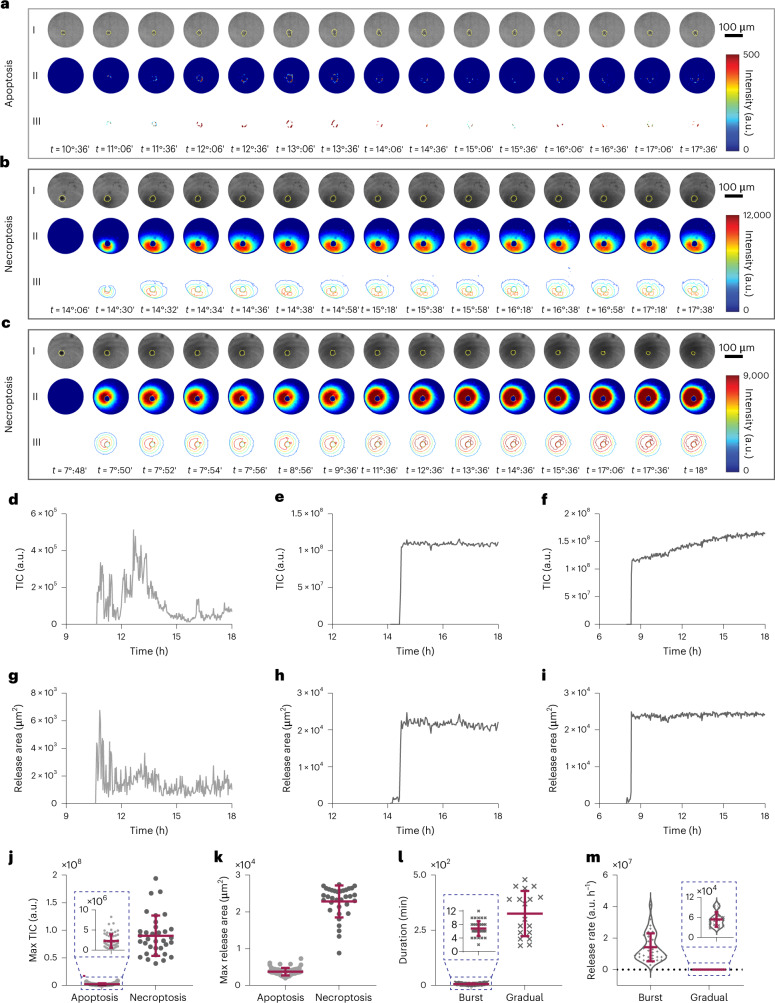
Visualization of content release during apoptosis and necroptosis. **a**–**c**, Representative 4D spatiotemporal release maps and contour plots along with the optical images at selected time points for an apoptotic cell (**a**) and two necroptotic ones (**b** and **c**). **d**–**f**, TIC curves associated with the amount of the released contents over time for the cells shown in **a**,**b** and **c**, respectively. **g**–**i**, Release area curves presenting the area occupied by the released products during cell death on the sensor surface over time for the cells shown in **a**,**b** and **c**, respectively. **j**,**k**, 1D scatterplots indicating the maximum TIC and areal coverage by the released products for 66 and 34 apoptotic and necroptotic cells, respectively. **l**, 1D scatterplot comparing the duration of the burst and gradual release for the necroptotic cells. **m**, Release rates of the necroptotic cells during the burst and gradual periods. In total, 191 and 186 single-cell measurements were performed for the apoptotic and necroptotic populations, respectively. The error bars in **j**, **k**, **l** and **m** represent the mean ± s.d. of dead cells from these populations, with *n*= 66 for apoptotic cells and *n* = 34 for necroptotic cells. The dashed blue boxes are used to better visualize the small changes in some of the parameters.

As shown by fluorescence imaging (Supplementary Figs. 11 and 12), the K562 cells experienced both apoptosis and necroptosis following established treatment protocols (see Methods for details). Next, we used the system with unfunctionalized plasmonic substrates to capture the spatiotemporal signature of each death type resulting from the binding of all the released contents to the gold nanohole arrays. Due to the rapid dynamics of release, especially during necroptosis, we performed the experiments with a 2 min time interval for 18 h. Figure 5a shows representative spatiotemporal release maps at selected time points for an apoptotic cell (Supplementary Videos 17 and 18). The optical images (first row in Fig. 5a) indicate the morphological characteristics of apoptosis including membrane blebbing and apoptotic bodies formation. The corresponding release maps and contour plots (second and third rows in Fig. 5a) show a local content release in the vicinity of the dying cell with a symmetric distribution. This observation agrees with the fact that apoptosis is usually considered as immunologically silent^56^.

Figure 5b,c show spatiotemporal analysis along with optical images for two representative necroptotic cells at selected time points (Supplementary Videos 19–22). Cell swelling followed by membrane rupture and cellular collapse, as morphological characteristics of necroptosis, can be verified by the optical images. In sharp contrast to the apoptotic cell, the spatiotemporal release maps and contour plots indicate a very rapid release of massive cell content (within ~10 min) upon membrane permeabilization that spreads over a large area around the cells. This bursting behaviour in necroptosis, which ejects biomolecules to longer distances, might mediate long-distance communication between dying and immune cells and increase the immunogenicity of necroptosis.

To quantify the differences between apoptosis and necroptosis release patterns, we extracted the TIC and release area curves. We observed that necroptotic cells presented themselves in two different ways. The cell shown in Fig. 5b exhibited strong variations in intensity (Fig. 5e) and release area (Fig. 5h) only at the time of the burst (~14:30 time point) due to cell membrane disintegration. Its corresponding spatiotemporal release maps and contour plots indicate that the burst followed an asymmetric pattern, probably due to the non-uniform pore formation on the membrane. The bursting behaviour of the cell shown in Fig. 5c also indicated an asymmetric release pattern initially. Interestingly, it experienced a resting period of around 40 min after the burst (~7:50 time point), followed by a gradual release of biomolecules that lasted around 7 h (Fig. 5f). This correlates well with recent studies showing that necroptotic cells keep synthesizing cytokines and chemokines after losing membrane integrity^57,58^. The release maps and contour plots (Fig. 5c) together with the corresponding TIC (Fig. 5f) and release area curves (Fig. 5i) highlight that the gradual release might affect the close vicinity of the cell in a symmetric pattern. The differences in the duration and areal coverage between burst and gradual release during necroptosis hint that these two ways of release may relate to or even instigate different immune responses in the cell vicinity and distant sites where they are sensed by the immune cells. It should also be noted that the apoptotic cell shown in Fig. 5a had notably less content release with smaller areal coverage compared with the necroptotic ones (Fig. 5d,g).

To evaluate the abovementioned behaviours for a larger population of single cells, we analysed 191 and 186 cells for apoptosis and necroptosis, respectively. Figure 5j compares the maximum TIC values associated with the amount of the released products for the two populations, indicating a ~40-fold intensity change for the necroptotic ones. Similarly, the maximum areal coverage of the necroptotic cells was six times higher than that of their apoptotic counterparts (Fig. 5k). To obtain more details about the release kinetics, we extracted the release rates and durations from the TIC curves. Of note, the high variability of the mentioned curves for the apoptotic cells (Fig. 5d,g) limits the extraction of such parameters.

For the necroptotic cells, the duration of the burst (6.8 min on average, Fig. 5l) was around 48 times shorter than that of gradual release (325.8 min on average, Fig. 5l). The release rate of the burst was 250 times faster than that of the gradual one (Fig. 5m). Consequently, this massive content release within a short time during the burst period might be attributed to the involvement of necroptosis at the onset of certain cancer types to trigger proper immune responses. On the other hand, the slow and local release during the gradual period for a long time could be used as a long-term signal for cell debris clearance.

### IgG secretion in human PBMCs

Successful single-cell analysis of a large number of hybridomas, which revealed different secretion profiles and provided insights into secretion kinetics, suggested that we can apply the system to study clinical samples. For this purpose, we investigated a population of human PBMCs to analyse antibody-secreting cells (ASCs). These cells circulate in the peripheral blood in response to infection or vaccination and then migrate to the bone marrow. They are called long-lived plasma cells and maintain the secretion of antibodies^59^. Due to their rarity and functional diversity^60^, monitoring them at single-cell resolution can provide valuable insight into their roles in various immune scenarios.

**Fig. 6:**
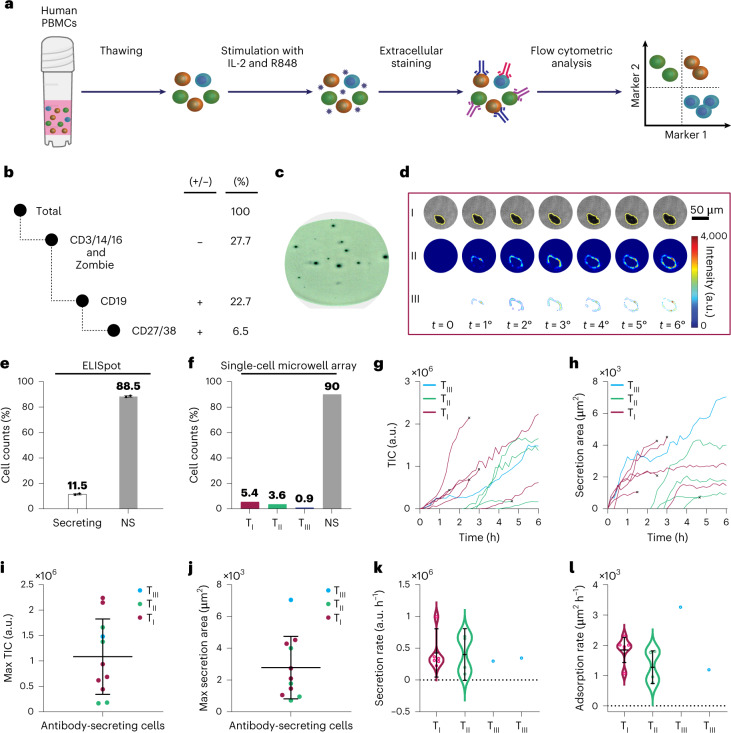
IgG secretion monitoring in ex vivo stimulated PBMCs. **a**, Schematic of PBMCs stimulation and staining followed by flow cytometric analysis for ASC enrichment. **b**, Summary of the cell sorting assay indicating that a small fraction (6.5%) of the ex vivo stimulated PBMCs were memory B cells differentiated into ASCs. **c**, A representative ELISpot result for the sorted cells. **d**, A representative 4D spatiotemporal secretion map for an ASC obtained by the plasmonic single-cell microwell array system showing secretion kinetics and morphological changes at the single-cell resolution. **e**,**f**, Secretion percentage in a population of ASCs analysed by ELISpot (**e**) and the single-cell microwell array system (**f**). With the plasmonic microwell array, 9.9% of the cells are found to be secreting, consistent with the results obtained by the ELISpot assay (11.5%). Error bars represent the s.d. between two replicates for each column (Extended Data Fig. 1). **g**,**h**, TIC (**g**) and secretion area curves (**h**) showing different secretion type ASCs in the population after monitoring them for 6 h. Each curve describes the changes for a single cell, and ‘×’ denotes the apoptosis onsets. **i**,**j**, 1D scatterplots indicating maximum TIC (**i**) and area covered by the secretion (**j**). The large s.d.s indicate the heterogeneous nature of the population (see Supplementary Table 2 for details). **k**,**l**, Secretion (**k**) and adsorption rates (**l**) of IgGs for the three types of ASC in the population, illustrating wide distributions for the types I and II ASCs. In total, 111 single-cell measurements were performed from the sorted PBMC population. The error bars in **i**, **j**, **k** and **l** represent the mean ± s.d. of secreting cells from this population, with *n* = 11 (6, 4 and 1 in the T1, T2 and T3 groups, respectively).

**Extended Data Fig. 1:**
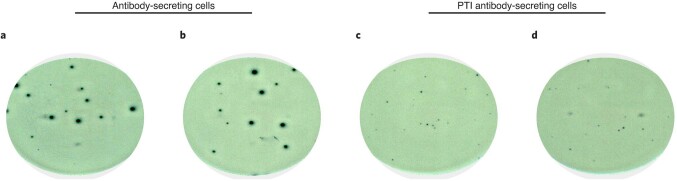
ELISpot assay for human IgG detection in antibody-secreting cells. **a** and **b**, Two repetitions of IgG detection using ELISpot for memory B cells from PBMCs that were ex vivo differentiated into antibody-secreting cells. A population of 100 cells was incubated in each well for six hours. 11 cells in (a) and 12 cells in (b) were found secreting. c and d, IgG detection in PTI antibody-secreting cells as a control experiment. No secreting cell was observed.

**Extended Data Fig. 2:**
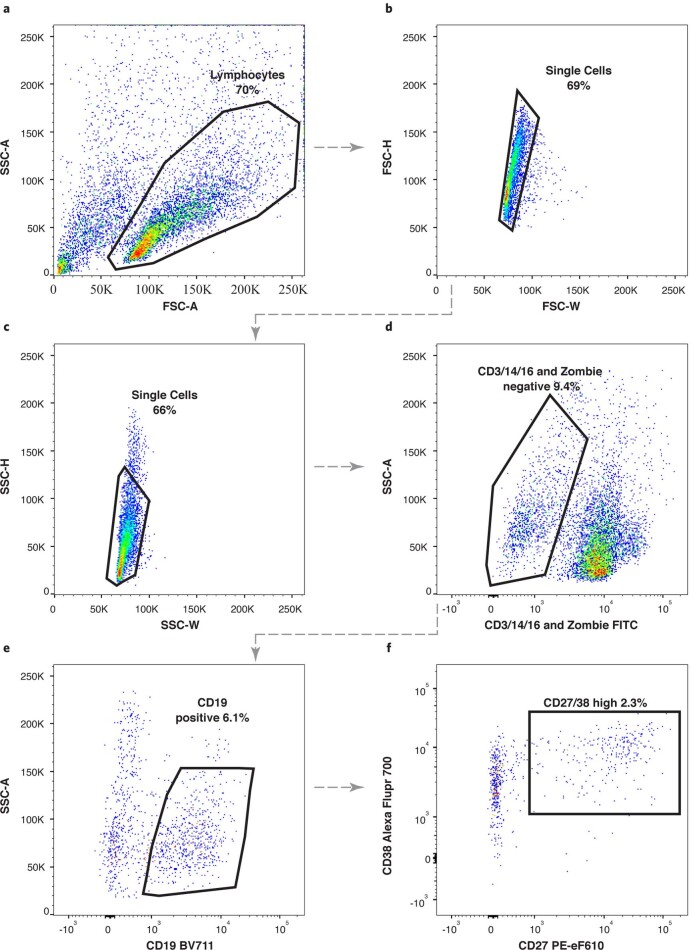
Flow cytometric analysis of human PBMCs. **a**, Excluding cell debris and selecting lymphocytes in the population based on forward and side scatters. **b** and **c**, Excluding doublets and selecting singlets based on forward and side scatters, respectively. **d**, Live cells (Zombie negative) were gated on CD3/14/16 (negative) to exclude the monocytes, T and natural killer cells. **e**, Gating the obtained population on CD19 (positive) to reach the B cells. **f**, Within the B cells, antibody-secreting cells were identified as CD27/38 (high) cells. Arrows show the gating strategy for detecting the potentially antibody-secreting cells.

**Extended Data Fig. 3:**
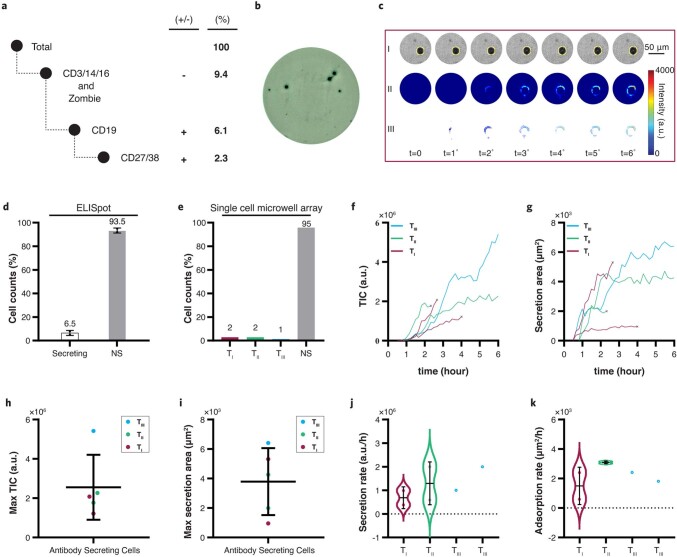
IgG secretion in ex vivo stimulated PBMCs. **a**, Summary of the cell sorting assay indicating a small fraction (2.3%) of the ex vivo stimulated PBMCs were memory B cells differentiated into ASCs. **b**, A representative ELISpot result for the sorted cells that allows counting the number of secreting cells. **c**, A representative 4D spatiotemporal secretion map for an ASC obtained by the plasmonic single-cell microwell array platform showing secretion kinetics and morphological changes at single-cell resolution. **d** and **e**, Secretion percentage in a population of ASCs analyzed by ELISpot and the single-cell microwell array platform, respectively. Error bars represent the standard deviation between two replicates for each column. **f** and **g**, TIC and secretion area curves illustrating different secretion types in the population after monitoring them for six hours (TI: type I, TII: type II, and TIII: type III). Each curve describes the changes for a single cell, and × shows the apoptosis onsets. **h** and **i**, One-dimensional scatter plots indicating maximum TIC and area covered by the secretion. j and k, Secretion and adsorption rates of IgGs for the three types of ASCs in the population. In total, 100 single-cell measurements were performed from the sorted PBMC population. The error bars in **h**, **i**, **j** and **k** represent the mean ± s.d. of secreting cells from this population, with *n* = 5 (2, 2 and 1 in the T1, T2 and T3 groups, respectively).

Given the low number of circulating ASCs in the peripheral blood, we stimulated ex vivo memory B cells from PBMCs, which then differentiated into ASCs^61^ (Fig. 6a). ASCs express the B cell marker CD19, and high levels of CD27 and CD38. We used these markers to sort the ASCs, which are 6.5% of the total population (Fig. 5b and Supplementary Fig. 13). Flow cytometry is limited to providing information about the cells that can potentially secrete IgGs. In other words, the obtained result does not necessarily correspond to the percentage of the cells that are actually secreting. For a more complete characterization, we analysed the sorted cells by ELISpot and our system. Their representative results after 6 h of monitoring are presented in Fig. 6c and d, respectively (Extended Data Fig. 1 and Supplementary Videos 23 and 24). ELISpot results indicated that only 11.5% of the sorted ASCs actually secreted IgGs (Fig. 6e). In line with these results, our system showed a 9.9% secretion frequency from a population of 111 sorted ASCs (Fig. 6f). We also subjected the plasmonic single-cell microwell array to a second PBMC sample from another healthy donor (Extended Data Figs. 2 and 3 and Supplementary Videos 25 and 26) with a lower frequency of the secreting cells (6.5 % of the ASCs based on ELISpot and 5% with single-cell microwell array), illustrating the capability of our system to capture a few secreting cells in hundreds of ASCs.

The spatial distribution of the secreted IgG molecules for these cells can also be investigated using the secretion maps and contour plots. For instance, in Fig. 6d, the secretion adopted an asymmetric C-shape pattern around the cell during the first 3 h and became more uniform by the end of the observation time. To gain additional insights into the spatiotemporal secretory behaviour of ASCs, we show TIC and secretion area curves in Fig. 6g,h. We observed that this cell population also has the three distinct secretion profiles, with 5.4%, 3.6% and 0.9% for types I, II and III, respectively. Figure 6i,j show the maximum TICs and secretion areas for the three types of secreting cell. In addition, we extracted the secretion kinetics by calculating the secretion and adsorption rates from Fig. 6g,h through linear curve fitting (results are given in Fig. 6k,l, respectively). The large standard deviations (see Supplementary Table 2 for more details) in all four plots indicate that the population is highly heterogeneous. The comprehensive information on secretion dynamics endowed by the plasmonic single-cell microwell array enables a multiparametric analysis for a patient-derived sample—an advantage over conventional methods. For applications where the frequency of the target cells is very low, such as monitoring antigen-specific ASCs^62^, combining FACS-based enrichment with our system could be more powerful than using either one of them alone as they complement each other.

## Outlook

**Extended Data Fig. 4:**
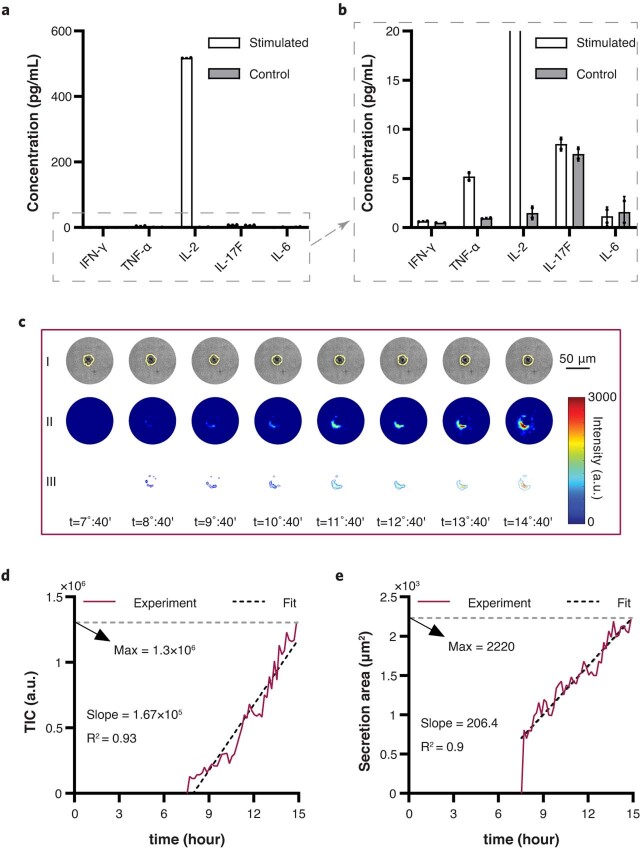
Spatiotemporal analysis of IL-2 secretion from mouse EL-4 cells. **a** and **b**, Characterization of secreted cytokines by the EL-4 cells using LEGENDplex™ kit. (b) zooms in to show the result for the cytokines with a lower level of secretion compared with IL-2. Error bars represent the standard deviation between two replicates for each column. **c**, A 4D spatiotemporal secretion map for a representative secreting EL-4 cell obtained by the plasmonic single-cell microwell array platform illustrating the secretion kinetics and morphological changes. (I) are the time-lapse optical images, (II) are the spatiotemporal secretion maps, and (III) are the secretion contour plots. **d** and **e**, TIC and secretion area curves indicating the amount of IgGs secreted over time and the area covered by the secretion, respectively. Linear curve fitting is used to extract the secretion and adsorption rates (the slopes). The maximum amount of secretion and area covered by the secretion (Max) are defined with the gray dashed lines.

By simultaneously visualizing the spatiotemporal distribution of released factors and monitoring cell morphology and motility over a long observation time at a temporal resolution of minutes from hundreds of individual cells, the label-free plasmonic single-cell microwell array can provide new insights into biological processes. The biosensor substrate can be adapted to detect different secretions, including cytokines, extracellular vesicles and antibodies. It is important to consider that secretion rates of such secretory species can vary largely. To illustrate the generality of the system for other biomolecules and to investigate its applicability to detect targets with lower secretion rates and smaller molecular weights compared to IgG^63^, we evaluated EL-4 cells to detect Interleukin-2 (IL-2), a key cytokine for the homoeostasis and differentiation of T lymphocytes^64^ (see Supplementary Discussion 6 and Extended Data Fig. 4). To increase the sensitivity of the system and consequently its ability to identify a wider range of biomolecules, optical resonance characteristics could be further optimized by engineering the nanostructure design. However, low-cost and wafer-scale fabrication of the substrates should be taken into account to maintain high-throughput screening.

Another aspect is the multiplexed detection of several secreted biomolecules simultaneously, which is essential for a more comprehensive analysis of cellular communication. However, this will require the implementation of functionalization methods enabling the immobilization of the corresponding receptors at spatially district locations on the substrate. The open-top configuration of the microwells used in this system enables convenient access for introducing various treatments during the experiments, for investigating their impacts on individual cell secretions, morphology and motility, and ultimately for retrieving the cells of interest via standard micromanipulators for downstream analyses. Furthermore, the capability to deterministically load a specific number of cells in each microwell, either similar or different cell types, along with machine-learning-assisted cell tracking can allow one to investigate secretion-mediated cell-signalling mechanisms. This could significantly facilitate the study of secretomes for which cell–cell contact is crucial, as in cancer–immune-cell interactions. The versatility and performance of the system and its compatibility with both adherent and non-adherent cells suggest that it can pave the way towards gaining a comprehensive understanding of single-cell secretory behaviours for applications ranging from basic research to drug discovery and personalized cell therapy.

## Methods

### Materials

PEGylated alkanethiols HS-C6-EG3OH, HS-C11-EG4OCH2COOH and HS-C11-EG3-biotin were obtained from Prochimia Surfaces. *N*-(3-Dimethylaminopropyl)-*N*′-ethylcarbodiimid-hydrochlorid (EDC, 25952-53-8), anhydrous sodium acetate (127-09-3), MES hydrat (1266615-59-1), ethanolamin (141-43-5), Ethylenediaminetetraacetic acid (EDTA, 60-00-4), bovine serum albumin (BSA, 9048-46-8), Toll-like receptor 7/8 agonist (R848, 144875-48-9), BM Condimed H1 (11088947001) and shikonin (54-952-43-1) were purchased from Sigma-Aldrich. Roswell Park Memorial Institute 1640 Medium (RPMI, 61870036), Dulbecco’s modified Eagle medium (DMEM, 41966029 and 218850025), fetal bovine serum (FBS, 10270106), phosphate buffered saline (PBS, 10010023), penicillin-streptomycin (15140122), *N*-Acetyl-l-cystein (NAC, 616-91-1), Pierce recombinant protein A/G (21186), *N*-2-hydroxyethylpiperazine-*N*-2-ethane sulfonic acid (HEPES, 15630056), Trypan blue solution (15250061), *β*-mercaptoethanol (21985023), AlexaFluor700 anti-human CD38 (56-0389-42), PE-eF610 anti-human CD27 (61-0279-42), Vivid Green Live/dead fixable stain kit (L34970), cell stimulation cocktail (00-4970-93), protein transport inhibitor cocktail (00-4980-93), cell viability imaging kit (R37609), CellEvent Caspase-3/7 Green Detection reagent (C10723) and ethidium homodimer-1 (E1169) were obtained from Thermo Fisher. Sulfo-*N*-hydroxysuccinimide (sulfo-NHS, 106627-54-7) and recombinant human TNF alpha protein (ab9642) were purchased from Abcam. SM-164 was obtained from LubioScience (HY-15989). Mouse and human IgG ELISpot kits (3825-2A and 3850-2H) and biotinylated mouse IL-2 antibody (3441-6-250) were purchased from Mabtech. FITC anti-human CD16 (302005), FITC anti-human CD14 (301803), FITC anti-human CD3 (300405), Brillant Violet 711 anti-human CD19 (302245) and LEGENDplex kits (N.741044 and N.740795) were purchased from Biolegend. Recombinant human IL-2 was obtained from Peprotech (200-02).

### Optical setup

We employed an inverted microscope (Nikon Ti-E) as the main optical platform for label-free cell secretion analysis, which is equipped with a customized microscope cell incubator (Life Imaging Services). For plasmonic intensity imaging measurements, we used a collimated near-infrared LED (Thorlabs, M850L3-C5) controlled by an LED driver (Thorlabs, LEDD1B) to achieve a narrowband illumination. To obtain maximal imaging quality at near-infrared spectrum, we took advantage of an sCMOS camera (Photometrics IRIS-15) with a quantum efficiency of >35% at 850 nm for EOT intensity acquisition. Besides, this camera had an active array of 5,056 × 2,960 pixels with a pixel size of 4.25 μm × 4.25 μm, which provided a large FOV (~1.1 mm × 0.65 mm) when combined with a medium power ×20 objective used for imaging. The microscope stage was controlled by software (Nikon Advanced Research) that enabled scanning different FOVs for high-throughput imaging.

### Fabrication of the plasmonic nanohole array substrates

To manufacture the gold nanohole array substrates, we took advantage of DUV lithography that provides wafer-scale and low-cost fabrication of the sensor chips on a transparent 4-inch fused silica substrate. In brief, after RCA cleaning of a 4-inch fused silica wafer, thin layers of Ti (10 nm) and Au (120 nm) were deposited on the wafer by e-beam evaporation (Alliance Concept EVA 760). Ti is not only used as the adhesion layer, but it also quenches the unwanted plasmonic modes at the interface of the Au and the wafer. Thus, the EOT signals are mainly created between the medium and the Au layer and are used to monitor the binding of secretory species to the gold surface. Then, we spin coated a layer of photoresist on the wafer and patterned an array of periodic nanoholes on the surface using a DUV stepper (ASML PAS5500/300). Next, we developed the pattern and transferred it onto the gold surface by ion beam etching (Oxford Instruments PlasmaLab 300). Finally, a resist removal step was done by oxygen plasma. In addition to the DUV lithography process, alternative wafer-scale nanofabrication methods such as nanoimprint lithography^65^ and interference lithography^66^ can be well-suited to low-cost manufacturing of nanohole arrays.

### Preparation of the plasmonic nanohole array substrates

After coating the wafer with photoresist as a protection layer, we diced it into 1 cm × 1 cm chips. Next, we removed the photoresist by immersing the chips in the resist stripper for 20 min in a 70 °C ultrasonic bath (twice), followed by an oxygen plasma treatment. Lastly, an RCA cleaning was performed to ensure removal of all polymer residues.

### Surface functionalization for IgG detection

We first cleaned the substrate by sequential washing with acetone, isopropanol and milli-Q water, followed by a 20 min UV treatment. Then a self-assembled monolayer (SAM) was formed on the surface by immersing the clean chip in COOH/OH-functional PEGylated alkanethiols at a 1:4 ratio and 2 mM final concentration of [-SH] in absolute ethanol overnight at room temperature. Next, the chip was washed with ethanol (three times, each for 5 min) to remove the unbound thiols, rinsed with milli-Q water and dried with pressurized nitrogen. After that, we activated the carboxylic group by immersing the chip in a cross-linking solution comprising EDC (200 mM) and sulfo-NHS (50 mM) in MES buffer for 20 min at room temperature. Upon activation, such groups are ready to bind to protein A/G and stabilize the biorecognition layer. Next, the chip was incubated in protein A/G (50 μg ml^−1^) solution diluted in acetate buffer for 2 h at room temperature on a thermomixer. The unreacted COOHs were deactivated by immersing the substrate in 1 M ethanolamine solution for 5 min at room temperature. We used 1% BSA to block the excess protein-binding sites for 1 h at room temperature. Finally, after rinsing the substrate with milli-Q water, it was ready for measurement. For the control experiment with hybridoma cells in the absence of protein A/G, the PEG functionalized substrate was incubated in 1% BSA for 1 h at room temperature, rinsed with milli-Q water and dried with pressurized nitrogen to be ready for measurement.

### Surface functionalization for IL-2 detection

After cleaning the substrate as explained above, the SAM layer was formed by immersing the substrate in biotin/OH-functional PEGylated alkanethiols at a ratio of 1:9 and 2 mM final concentration in absolute ethanol overnight at room temperature. Next, the substrate was washed with ethanol (three times, each for 5 min) to remove the unbound thiols, rinsed with milli-Q water and dried with pressurized nitrogen. After that, the substrate was treated with 50 μg ml^−1^ streptavidin solution diluted in PBS for 3 h at room temperature on a thermomixer. The substrate was then washed with PBS (three times, each for 5 min) to remove the unbound streptavidin and incubated in biotinylated mouse IL-2 antibody (50 μg ml^−1^) solution diluted in PBS for 2 h at room temperature on a thermomixer. After washing the substrate with PBS (three times, each for 5 min), 1% BSA solution was added to block the excess protein-binding sites for 1 h at room temperature. Finally, the substrate was rinsed with milli-Q water to be ready for measurement.

### SPR instrument and measurements

SPR measurements were performed using a commercial multiparametric SPR machine (MP-SPR Navi 210A VASA, Bionavis). The real-time sensograms were obtained by angular scanning (60°–75°) with a laser source at 670 nm wavelength and scan speed of 1.5 s. The measurements were done with a continuous buffer flow (PBS, 10 μl min^−1^) at room temperature. SPR sensors (purchased from Bionavis) were made of glass with 50 nm gold and 10 nm chromium coating as the adhesion layer. The sensor cleaning and SAM layer formation were done as explained for the plasmonic substrates. The rest of the functionalization steps were carried out in real time.

### Cell culture

Clonal mouse hybridoma cells (anti-CD45.1), mouse EL-4 cells (TIB-39, kindly provided by Dr Anne Wilson from the University of Lausanne) and K562 cells (CCL-243, obtained from ATCC) were cultured in high-glucose DMEM supplemented with 10% FBS, 10 mM HEPES, 100 U ml^−1^ penicillin and 100 μg ml^−1^ streptomycin at 37 °C and 5% CO_2_. The medium was refreshed, and the cells were split every 2–3 d.

Before each experiment, the viability of the cells was assessed using trypan blue staining to ensure that the cells were in good condition. For analysing the secretion of hybridoma and K562 cells, we modified the culture medium by using low-glucose DMEM and 300 μM NAC to reduce oxidative stress on the single cells and 1% BM Condimed (instead of 10% FBS) to reduce the non-specific products introduced by FBS while maintaining the cells’ viability. In the control experiments with hybridoma cells, to block the secretory pathway, a protein transport inhibitor cocktail (1x) containing brefeldin A and monensin was added to the medium. For analysing the mouse EL-4 cells, a stimulation cocktail (1x) was also added to the abovementioned medium. A viability kit was used according to the manufacturer’s protocol to evaluate the cells’ viability.

### Cell-death induction

To induce apoptosis and necroptosis, we followed the established protocols^67–69^. In brief, after spotting the K562 cells in the PDMS microwells, they were incubated in the cell culture medium (as described in the previous sections) supplemented with 50 ng ml^−1^ human TNF-α and 100 nM sm-164 for apoptosis induction. Necroptosis was triggered by treating the cells with 10 μM shikonin.

### Fluorescence imaging of different cell death types

To monitor apoptosis and necroptosis with fluorescence labelling, we incubated the treated cells (as described in the previous sections) with 5 μM CellEvent Caspase-3/7 Green Detection reagent and 1.6 μM ethidium homodimer-1, and captured the fluorescence images every 10 min. Apoptotic cells were expected to emit green fluorescence upon caspase-3/7 activation and red fluorescence after membrane permeabilization and binding of ethidium homodimer-1 to DNA. The necroptotic cells were expected to show only red fluorescence after membrane disintegration without caspase-3/7 signals.

### Generating the single-cell array on PDMS micromesh

We prepared an array of microwell structures by attaching a PDMS micromesh to the gold nanohole array chips to seed the single cells. The PDMS structures were fabricated on the basis of the standard photo-/soft-lithography procedure. Each microwell had a diameter of 200 μm and a height of 50 μm, generating a unit volume of 1.5 nl. We cleaned the PDMS device by sonication in 70% ethanol and dried it using pressurized nitrogen before cell seeding.

Single cells were isolated and dispensed into the microwell by employing the state-of-the-art cellenONE X1 technology (SCIENION). This technology uses a combination of a piezoelectric liquid dispenser and advanced image processing to enable deterministic dispensing of a large quantity of single cells. Each microwell was first prefilled with cell culture medium supplemented with 1% v/v glycerol to prevent liquid evaporation during the subsequent procedure. A suspension of the cells was centrifuged at 410 × *g* for 5 min and washed twice with serum-free media to remove the secreted materials. We adjusted the cell density to 2–3 × 10^5^ cells per ml and subsequently pipetted 50 μl of the suspension into a 384-well plate (the standard format for the dispenser). The piezoelectric voltage and pulse duration were set at 65 V and 48 μs, forming a droplet of 300 pl with cell culture media. Then, 10 μl of cell suspension was loaded into the dispensing nozzle, resulting in the seeding of single cells into microwells in a highly precise manner.

### Preparation and sorting of PBMCs

Venous blood was drawn from healthy donors at the Swiss Transfusion Center of Geneva, Switzerland, under its ethical approval. The volunteers were asked to read an information sheet for blood donation and to complete an online medical questionnaire on the day of donation. After the questionnaire was finalized, a PDF file was generated for printing and signed to give approval for blood donation.

Memory B cell stimulation was performed as detailed elsewhere^61^. Briefly, cryopreserved PBMCs from a blood donor were thawed and stimulated with R848 (1 μg ml^−1^) and human interleukin-2 (10 ng ml^−1^) in RPMI containing 10% FBS, 10 mM HEPES, 100 U ml^−1^ penicillin, 100 μg ml^−1^ streptomycin and 50 mM *β*-mercaptoethanol. The cells were incubated (37 °C and 5% CO_2_) for 5 d, collected and washed with PBS. Next, the cells were live/dead stained with Vivid Green (1/5,000 in PBS) for 20 min at 4 °C. After washing the cells with PBS and removing the supernatant, the cells were stained in sorting buffer (PBS, 50 μM EDTA and 0.2% BSA) using a panel of fluorescent antibodies designed for isolation of antibody-secreting cells including FITC anti-human CD16 (1:200), FITC anti-human CD14 (3:500), FITC anti-human CD3 (1:100), Brillant Violet 711 anti-human CD19 (1:200), Alexa Fluor 700 anti-human CD38 (1:50) and PE-eF610 anti-human CD27 (1:50) for 20 min at room temperature. Finally, the cells were washed with PBS, resuspended in sorting buffer and sorted using FACSAria II instrument (BD Biosciences) at 4 °C. After sorting, the cells were incubated at 37 °C for 3 h in the mentioned medium for full recovery and in preparation for the secretion analysis using the single-cell plasmonic microwell array and ELISpot assay.

### ELISpot assays for mouse hybridomas and human ASCs

We first activated the polyvinylidene fluoride or polyvinylidene difluoride (PVDF) plates using 15 μl per well of 35% ethanol for 20 s and then washed the wells five times with milli-Q water. Following the manufacturer’s recommended protocol, the plates were treated with 100 μl of the 15 μg ml^−1^ capture antibodies (anti-IgG antibody for mouse IgG and MT91/145 antibody for human IgG) at 4 °C overnight. Next, we washed the wells five times with BPS, and for blocking, we incubated them with the medium used for culturing the cells for 1 h at room temperature. After that, the cells (mouse hybridomas and human ASCs) were counted and seeded in the wells in duplicate. For the control wells, the secretory pathway was blocked by adding the protein transport inhibitor cocktail (1×). After incubation at 37 °C in a humidified incubator (12 h for mouse hybridomas and 6 h for human PBMCs), we removed the cells and washed the plate with PBS (five times). Of the 1 μg ml^−1^ detection antibodies (anti-IgG-biotin for mouse IgG and MT78/145-biotin for human IgG), 100 μl was added to each well for 2 h at room temperature, followed by washing with PBS (five times). Then, streptavidin-ALP (for mouse IgG) and streptavidin-HRP (for human IgG) were diluted in PBS (1:1,000) and added to the wells (100 μl) for 1 h at room temperature. To develop the spots, we washed the plates with PBS (five times) and added 100 μl per well of the substrate solutions (BCIP/NBT for mouse IgG and TMB for human IgG). When the spots emerged, we stopped the development by washing the plates with deionized water. Finally, the spots were counted using Bioreader-6000-E (BIO-SYS).

### Cytokine quantification for EL-4 and K562 cells

A panel of cytokines was quantified for the EL-4 and K562 cells using LEGENDplex kits according to the manufacturer’s recommendation. In brief, 2 × 10^4^ EL-4 cells in 500 μl medium were treated with a stimulation cocktail containing phorbol 12-myristate 13-acetate (PMA) and ionomycin (1×) for 15 h. A group of non-stimulated cells was used as a control. For the K562 cells, 10^4^ cells were cultured in 500 μl medium for 24 h without any stimulation. Then, the cell supernatants were collected and incubated with antibody-conjugated beads. Next, the beads were washed, and the biotinylated detection antibodies were added to form the sandwich immunocomplex. Finally, streptavidin-phycoerythrin was added and the fluorescence intensity was measured using a Gallios flow cytometer (Beckman Coulter). Data analysis was done using the LEGENDplex software (v8.0).

### Machine-learning-assisted image processing

The 4D spatiotemporal secretion maps with cell tracking were generated using an in-house code, written and executed in a MATLAB live script that combines conventional MATLAB code and formatted text.

The raw images showed subfields of the single-cell microwell array, which contain individual cells and the empty reference wells. A time lapse of these images (in.tif or.tiff image file formats) was loaded and sorted as variables in the MATLAB workspace. The raw inputs were cropped (350 × 350 pixels) to analyse each specific cell-spotted sensing microwell and its empty counterpart.

### Cell tracking

Cropped but otherwise unprocessed images of the cell-spotted sensing microwells were semantically segmented into ‘cell’ and ‘background’ using ilastik^70^, an interactive machine-learning toolkit for bioimaging, in headless mode. A random set of approximately 100 images from both different reference and cell-spotted sensing microwells (including cells that underwent mitosis) in different time instances were used to manually train the algorithm using the pixel classification workflow of the toolkit. ilastik was used to select and compute six features for each pixel (Gaussian, Laplacian of Gaussian, Gaussian gradient magnitude, difference of Gaussians, dtructure tensor eigenvalues and Hessian of Gaussian eigenvalues), with seven kernel radiuses for Gaussian (0.3 pixel, 0.7 pixel, 1.0 pixel, 1.6 pixels, 3.5 pixels, 5.0 pixels and 10.0 pixels) and six kernel radiuses (0.7 pixel, 1.0 pixel, 1.6 pixels, 3.5 pixels, 5.0 pixels and 10.0 pixels) for the other features, yielding a vector of 37 values for each pixel. For training, subsets of the pixels were labelled as ‘cell’ or ‘background’ by a human. The identity of the remaining pixels was predicted using a random forest classifier with 100 trees. After training and batch processing of images, the classifier outputted a respective probability map with pixel values ranging from 0 (‘cell’) to 1 (‘background’). The probability map images were smoothed with an arithmetic mean filter and binarized using Otsu’s method, thus obtaining a binary ‘cell’-‘background’ mask for each frame in the time lapse. To identify (and exclude from subsequent analysis) all the spatiotemporal positions occupied by the motile cell, a cumulative binary mask was built such that each image was the union of the frames preceding it.

### Analysis of spatiotemporal secretion profile

The secretion profile of single cells was extracted through the creation of new vectors for both the microwell images (reference and sensing) and the cumulative binary masks. The new vectors corresponded to the pixel-wise absolute difference between the first image and all subsequent images of the time lapse, as Δ*I*(*t* + 1) = |*I*(*t* + 1) − *I*(1)|, where *I* and *t* are pixel intensity and time, respectively. The ‘difference images’ of the microwells were smoothed using a Gaussian kernel with standard deviation *σ* = 1.5.

Background and baseline drift correction took place by selecting a small region of interest (ROI) in the first ‘difference image’ of the reference microwells and propagating it along the time lapse. The average grey-level intensity for each reference ‘difference image’ was calculated using *μ*_signal + 3*σ*_signal, where *μ* is the average grey-level intensity of the ROI and *σ* is the known standard deviation. The resulting value was pixel-wise subtracted from the corresponding cell-spotted sensing microwell ‘difference image’.

These preliminary secretion maps were further refined by using the circular Hough transform to identify the border of the microwell and exclude the area outside of it. Additionally, a so-called ‘secretion ROI’ was selected by a human in the sensing microwell ‘difference images’ to reduce noise due to edge artefacts and intensity differences within the microwell but far from (and therefore unrelated to) the cell. An iterative *K*-means clustering algorithm was optionally used to remove the intensity difference clusters, which were small and far away from the centroid of the cell. Finally, the secretion maps were multiplied by the ‘difference image’ of the cumulative mask to completely remove the intensity changes occurring due to the presence and movement of the cell.

### Secretion analysis

Two metrics were developed to quantify the secretion profile of single cells in real time. The first metric is termed absolute intensity change (TIC), which was calculated as the total intensity of the non-zero pixels in each secretion map frame. The second metric is the secretion area, defined as the area occupied by the binding of secreted materials to the capture probes on the functionalized chip surface, which was calculated by multiplying the number of non-zero pixels in the secretion map by the physical area of each pixel.

### Kinetics modelling

The onset of secretion was defined as the first instance when the TIC of the background-corrected ‘difference image’ was greater than zero. To extract kinetic information from the TIC and secretion area curves, we used linear curve fitting to calculate the secretion and absorption rates. We fitted linear equations to the curves for the time interval between the onset of secretion and the point when the signals reached 80% of the maximum experimental values. It should be noted that for the secreting cells type III and necroptotic cells with burst and gradual release, two equations were fitted to the linear parts. For this purpose, the plateau duration was defined as the time interval between the time point when the signals did not increase in three successive time frames and the moment when the signals experienced a further rise in three successive time frames. By doing so, we obtained two intervals for the fitting: from the onset of secretion to the point when the signals reached 80% of the average of the plateau, and from the end of the plateau to the moment when the signals reached 80% of the maximum experimental value. The coefficients of determination were greater than 0.9 when employing least-squares regression analyses to find the best fits.

Duration of the secretion was defined as the time interval when the linear fitting was valid for the TIC curves. For the secreting cells type III, the sum of both time intervals for the linear parts was considered as the duration of secretion.

### Statistics

Data were analysed using MATLAB 2019, Microsoft Excel 2019, GraphPad Prism, ilastik (v1.3.2), FlowJo (v10.8.0) and ImageJ (v1.53f51). The imaging data were collected using NIS-Elements Advanced Research. The data analysis for cytokine quantification using the LEGENDplex kits was conducted by the LEGENDplex software (v8.0). For analysing the populations, data were expressed as means ± s.d. Sample sizes are listed in each section along with the corresponding experiments.

### Reporting summary

Further information on research design is available in the Nature Portfolio Reporting Summary linked to this article.

## Supplementary information

Supplementary Information Supplementary discussion, figures, tables and references.

Reporting Summary

Supplementary Video 1 Time-lapse optical imaging of a type I hybridoma cell.

Supplementary Video 2 4D spatiotemporal secretion map for a hybridoma cell with a linear profile.

Supplementary Video 3 Time-lapse optical imaging of a type II hybridoma cell.

Supplementary Video 4 4D spatiotemporal secretion map for a hybridoma cell with a half-sigmoid profile.

Supplementary Video 5 Time-lapse optical imaging of a type III hybridoma cell.

Supplementary Video 6 4D spatiotemporal secretion map for a hybridoma cell showing a combination of the half-sigmoid and linear profiles.

Supplementary Video 7 Time-lapse optical imaging of a hybridoma cell monitored with a dense microwell array.

Supplementary Video 8 4D spatiotemporal secretion map for a hybridoma cell monitored with a dense microwell array.

Supplementary Video 9 Time-lapse optical imaging of a non-secreting mother cell.

Supplementary Video 10 4D spatiotemporal secretion map for a non-secreting mother cell.

Supplementary Video 11 Time-lapse optical imaging of secreting daughter cells following a linear pattern.

Supplementary Video 12 4D spatiotemporal secretion map for secreting daughter cells following a linear pattern.

Supplementary Video 13 Time-lapse optical imaging of a secreting mother cell with a half-sigmoid profile.

Supplementary Video 14 4D spatiotemporal secretion map for secreting a mother cell following a half-sigmoid pattern.

Supplementary Video 15 Time-lapse optical imaging of daughter cells with a linear secretion profile.

Supplementary Video 16 4D spatiotemporal secretion map for daughter cells with a linear secretion profile.

Supplementary Video 17 Time-lapse optical imaging of an apoptotic cell.

Supplementary Video 18 4D spatiotemporal release map for an apoptotic cell.

Supplementary Video 19 Time-lapse optical imaging of a necroptotic cell showing only bursting behaviour.

Supplementary Video 20 4D spatiotemporal release map for a necroptotic cell showing only bursting behaviour.

Supplementary Video 21 Time-lapse optical imaging of a necroptotic cell showing both bursting and gradual release patterns.

Supplementary Video 22 4D spatiotemporal release map for a necroptotic cell showing both bursting and gradual release patterns.

Supplementary Video 23 Time-lapse optical imaging of an ASC from the first healthy donor.

Supplementary Video 24 4D spatiotemporal secretion map for an ASC from the first healthy donor.

Supplementary Video 25 Time-lapse optical imaging of an ASC from the second healthy donor.

Supplementary Video 26 4D spatiotemporal secretion map for an ASC from the second healthy donor.

Supplementary Video 27 Time-lapse optical imaging of an EL-4 cell secreting IL-2.

Supplementary Video 28 4D spatiotemporal secretion map for an EL-4 cell secreting IL-2.

## Data Availability

The main data supporting the results in this study are available within the paper and its Supplementary Information. The raw and analysed datasets generated during the study are too large to be publicly shared, yet they are available for research purposes from the corresponding author on reasonable request.

## Code availability

The custom MATLAB codes used for the spatiotemporal analyses are available from the corresponding author on reasonable request.

## References

[1] Duronio RJ, Xiong Y (2013). Signaling pathways that control cell proliferation. Cold Spring Harb. Perspect. Biol..

[2] Seldin MM (2018). A strategy for discovery of endocrine interactions with application to whole-body metabolism. Cell Metab..

[3] Eyer K (2017). Single-cell deep phenotyping of IgG-secreting cells for high-resolution immune monitoring. Nat. Biotechnol..

[4] Kawamoto Y, Nakajima Y, Kuranaga E (2016). Apoptosis in cellular society: communication between apoptotic cells and their neighbors. Int. J. Mol. Sci..

[5] Fogarty CE, Bergmann A (2015). The sound of silence: signaling by apoptotic cells. Curr. Top. Dev. Biol..

[6] Tanzer MC (2020). Quantitative and dynamic catalogs of proteins released during apoptotic and necroptotic cell death. Cell Rep..

[7] Huse M, Quann EJ, Davis MM (2008). Shouts, whispers and the kiss of death: directional secretion in T cells. Nat. Immunol..

[8] Murphy LO, Smith S, Chen RH, Fingar DC, Blenis J (2002). Molecular interpretation of ERK signal duration by immediate early gene products. Nat. Cell Biol..

[9] Huse M, Lillemeier BF, Kuhns MS, Chen DS, Davis MM (2006). T cells use two directionally distinct pathways for cytokine secretion. Nat. Immunol..

[10] Quann EJ, Merino E, Furuta T, Huse M (2009). Localized diacylglycerol drives the polarization of the microtubule-organizing center in T cells. Nat. Immunol..

[11] Uhlén M (2019). The human secretome. Sci. Signal..

[12] Ma C (2011). A clinical microchip for evaluation of single immune cells reveals high functional heterogeneity in phenotypically similar T cells. Nat. Med..

[13] Lu Y (2015). Highly multiplexed profiling of single-cell effector functions reveals deep functional heterogeneity in response to pathogenic ligands. Proc. Natl Acad. Sci. USA.

[14] Dewhurst JA (2017). Characterisation of lung macrophage subpopulations in COPD patients and controls. Sci. Rep..

[15] Zhu J, Paul WE (2010). Heterogeneity and plasticity of T helper cells. Cell Res..

[16] Wong IY, Bhatia SN, Toner M (2013). Nanotechnology: emerging tools for biology and medicine. Genes Dev..

[17] Paltridge JL, Belle L, Khew-Goodall Y (2013). The secretome in cancer progression. Biochim. Biophys. Acta.

[18] Hellinger JW (2020). Identification of drivers of breast cancer invasion by secretome analysis: insight into CTGF signaling. Sci. Rep..

[19] Pinho AG, Cibrão JR, Silva NA, Monteiro S, Salgado AJ (2020). Cell secretome: basic insights and therapeutic opportunities for CNS disorders. Pharmaceuticals.

[20] Bucheli OTM, Sigvaldadóttir I, Eyer K (2021). Measuring single-cell protein secretion in immunology: technologies, advances, and applications. Eur. J. Immunol..

[21] Donck FVer, Downes K, Freson K (2020). Strengths and limitations of high-throughput sequencing for the diagnosis of inherited bleeding and platelet disorders. J. Thromb. Haemost..

[22] Chen, Z., Chen, J. J. & Fan, R. Single-cell protein secretion detection and profiling. *Annu. Rev. Anal. Chem.* **12**, 431–449 (2019). 10.1146/annurev-anchem-061318-115055 30978293

[23] Hernandez-Fuentes MP, Warrens AN, Lechler RI (2003). Immunologic monitoring. Immunol. Rev..

[24] Saillard M, Cenerenti M, Romero P, Jandus C (2021). Impact of immunotherapy on CD4 T cell phenotypes and function in cancer. Vaccines.

[25] Choi, J. R. Advances in single cell technologies in immunology. *BioTechniques* **69**, 227–236 (2020). 10.2144/btn-2020-0047 32777935

[26] Kulkarni RP, Che J, Dhar M, Di Carlo D (2014). Research highlights: microfluidic single-cell analysis from nucleic acids to proteins to functions. Lab Chip.

[27] An X (2017). Single-cell profiling of dynamic cytokine secretion and the phenotype of immune cells. PLoS ONE.

[28] Love JC, Ronan JL, Grotenbreg GM, van der Veen AG, Ploegh HL (2006). A microengraving method for rapid selection of single cells producing antigen-specific antibodies. Nat. Biotechnol..

[29] Han Q (2012). Polyfunctional responses by human T cells result from sequential release of cytokines. Proc. Natl Acad. Sci. USA.

[30] Konry T, Golberg A, Yarmush M (2013). Live single cell functional phenotyping in droplet nano-liter reactors. Sci. Rep..

[31] Chokkalingam V (2013). Probing cellular heterogeneity in cytokine-secreting immune cells using droplet-based microfluidics. Lab Chip.

[32] Zhou, L., Chen, P. & Simonian, A. in *Biosensors. Current and Novel Strategies for Biosensing.* (Villarreal-Gómez, L. J. & Iglesias, A. L. eds) Chap. 9 (IntechOpen, 2021).

[33] Bocková, M., Slabý, J., Špringer, T. & Homola, J. Advances in surface plasmon resonance imaging and microscopy and their biological applications. *Annu. Rev. Anal. Chem.* **12**, 151–176 (2019). 10.1146/annurev-anchem-061318-115106 30822102

[34] Cunningham BT, Zhang M, Zhuo Y, Kwon L, Race C (2016). Recent advances in biosensing with photonic crystal surfaces: a review. IEEE Sens. J..

[35] Nguyen HH, Park J, Kang S, Kim M (2015). Surface plasmon resonance: a versatile technique for biosensor applications. Sensors.

[36] Zeng Y (2017). Recent advances in surface plasmon resonance imaging: detection speed, sensitivity, and portability. Nanophotonics.

[37] Oh S-H (2021). Nanophotonic biosensors harnessing van der Waals materials. Nat. Commun..

[38] Jackman JA, Ferhan AR, Cho N-J (2017). Nanoplasmonic sensors for biointerfacial science. Chem. Soc. Rev..

[39] Brolo AG (2012). Plasmonics for future biosensors. Nat. Photon..

[40] Giannini V (2010). Controlling light localization and light–matter interactions with nanoplasmonics. Small.

[41] Juan-Colás J, Hitchcock IS, Coles M, Johnson S, Krauss TF (2018). Quantifying single-cell secretion in real time using resonant hyperspectral imaging. Proc. Natl Acad. Sci. USA.

[42] Parray HA (2020). Hybridoma technology a versatile method for isolation of monoclonal antibodies, its applicability across species, limitations, advancement and future perspectives. Int. Immunopharmacol..

[43] Lu R-M (2020). Development of therapeutic antibodies for the treatment of diseases. J. Biomed. Sci..

[44] Arora S, Saxena V, Ayyar BV (2017). Affinity chromatography: a versatile technique for antibody purification. Methods.

[45] Kulkarni, P. et al. in *Gels Horizons: From Science to Smart Materials.* (Tripathi, A. & Melo, S. V. eds) 85–139 (Springer, 2021).

[46] Li J (2013). Human vascular endothelial cell morphology and functional cytokine secretion influenced by different size of HA micro-pattern on titanium substrate. Colloids Surf. B.

[47] Leuning DG (2018). The cytokine secretion profile of mesenchymal stromal cells is determined by surface structure of the microenvironment. Sci. Rep..

[48] Yeong FM (2013). Multi-step down-regulation of the secretory pathway in mitosis: a fresh perspective on protein trafficking. Bioessays.

[49] Valente C, Colanzi A (2015). Mechanisms and regulation of the mitotic inheritance of the golgi complex. Front. Cell Dev. Biol..

[50] Pagliuca FW (2011). Quantitative proteomics reveals the basis for the biochemical specificity of the cell-cycle machinery. Mol. Cell.

[51] Ly T (2014). A proteomic chronology of gene expression through the cell cycle in human myeloid leukemia cells. eLife.

[52] Lane KR (2013). Cell cycle-regulated protein abundance changes in synchronously proliferating HeLa cells include regulation of pre-mRNA splicing proteins. PLoS ONE.

[53] Okada M, Kusunoki S, Ishibashi Y, Kito K (2017). Proteomics analysis for asymmetric inheritance of preexisting proteins between mother and daughter cells in budding yeast. Genes Cells.

[54] Cooper S (2007). Membrane-elution analysis of content of cyclins A, B1, and E during the unperturbed mammalian cell cycle. Cell Div..

[55] Tanzer MC (2022). A proteomic perspective on TNF-mediated signalling and cell death. Biochem. Soc. Trans..

[56] Bertheloot D, Latz E, Franklin BS (2021). Necroptosis, pyroptosis and apoptosis: an intricate game of cell death. Cell. Mol. Immunol..

[57] Orozco SL (2019). RIPK3 activation leads to cytokine synthesis that continues after loss of cell membrane integrity. Cell Rep..

[58] Yatim N (2015). RIPK1 and NF-κB signaling in dying cells determines cross-priming of CD8^+^ T cells. Science.

[59] Carter MJ, Mitchell RM, Meyer Sauteur PM, Kelly DF, Trück J (2017). The antibody-secreting cell response to infection: kinetics and clinical applications. Front. Immunol..

[60] Good-Jacobson KL (2018). Strength in diversity: phenotypic, functional, and molecular heterogeneity within the memory B cell repertoire. Immunol. Rev..

[61] Eberhardt CS (2020). Persistence of varicella-zoster virus-specific plasma cells in adult human bone marrow following childhood vaccination. J. Virol..

[62] Ramirez K (2021). Heterofunctional particles as single cell sensors to capture secreted immunoglobulins and isolate antigen-specific antibody secreting cells. Adv. Healthc. Mater..

[63] Han Q, Bradshaw EM, Nilsson B, Hafler DA, Love JC (2010). Multidimensional analysis of the frequencies and rates of cytokine secretion from single cells by quantitative microengraving. Lab Chip.

[64] Boyman O, Sprent J (2012). The role of interleukin-2 during homeostasis and activation of the immune system. Nat. Rev. Immunol..

[65] Chen J (2009). Gold nanohole arrays for biochemical sensing fabricated by soft UV nanoimprint lithography. Microelectron. Eng..

[66] Chang TY (2011). Large-scale plasmonic microarrays for label-free high-throughput screening. Lab Chip.

[67] González-Flores D, Rodríguez AB, Pariente JA (2014). TNFα-induced apoptosis in human myeloid cell lines HL-60 and K562 is dependent of intracellular ROS generation. Mol. Cell. Biochem..

[68] Zhu K (2018). Necroptosis promotes cell-autonomous activation of proinflammatory cytokine gene expression. Cell Death Dis..

[69] Huang X, Chen Z, Ni F, Ye X, Qian W (2020). Shikonin overcomes drug resistance and induces necroptosis by regulating the miR-92a-1-5p/MLKL axis in chronic myeloid leukemia. Aging.

[70] Berg S (2019). ilastik: interactive machine learning for (bio)image analysis. Nat. Methods.

