# Supplementary material for: High-throughput spatiotemporal monitoring of single-cell secretions via plasmonic microwell arrays
Source: Nat Biomed Eng. 2023 Apr 3;7(7):943–58. doi: 10.1038/s41551-023-01017-1 (PMC10365996; doi:10.1038/s41551-023-01017-1)
Supplement: Supplementary file 1 — Supplementary discussion, figures, tables and references. [file 41551_2023_1017_MOESM1_ESM.pdf]

nature biomedical engineering

Article https://doi.org/10.1038/s41551-023-01017-1

High-throughput spatiotemporal monitoring of single-cell secretions via plasmonic microwell arrays

In the format provided by the authors and unedited

Supplementary Discussion 1 l Microwell array throughput and temporal resolution

In microwell array-based platforms, there is an inherent trade-off between throughput and temporal resolution. For our microwell array, the design parameters that influence the throughput include the diameter of the wells, the periodicity of the wells, the arrangement of the referencing, and the size of the plasmonic substrate. In the current configuration, we used 200 μm diameter circular wells, 300 μm periodicity, one reference well for each sensing well (50% reference occupancy), and 1 cm x 1 cm sized plasmonic substrates (Supplementary Fig. 1a). Based on the obtained results shown in Figs 2 and 3, the platform has the potential to screen a higher number of cells by considering these four design parameters as follows. 1) For the well size, we take into account the average cell diameter (~10–30 µm), the spatial extent of the secretion in the extracellular space (~30–50 µm), and the cell movement (~5–20 µm). These give us a minimum microwell diameter of around 100 µm for the spatiotemporal secretion analysis. 2) By decreasing the periodicity to 200 μm, in one FOV (1.1 mm x 0.65 mm), 15 microwells can be imaged simultaneously (Supplementary Fig. 1b). 3) Out of these 15 wells in a given FOV, only one well is enough for background correction, thus improving reference occupancy from 50% to 6.7%. 4) The plasmonic substrate size can be kept 1 cm x 1 cm. This dense microwell array configuration allows to achieve a higher throughput and screen around 2500 wells (2330 cells).

Another important aspect for secretion monitoring, which can be affected by increasing the throughput, is the temporal resolution. Several events can influence the temporal resolution of our platform such as image acquisition time, stage movement time from one FOV to the next, and the waiting time for the medium stabilization at each new stage position (e.g., due to the slashing of the medium with the stage movement). In the current experimental settings, image acquisition and stage movement take place in ~20 msec and 30 msec, respectively, whereas we wait one second at each new stage position for the stabilization. Accordingly, the temporal resolution is practically decided by this waiting time before each capture. Supplementary Fig. 1c indicates the trade-off between the throughput and temporal resolution for both the current and dense microwell designs. At the full capacity, the dense microwell array allows screening around 2500 wells (2330 cells) at a 5.5-minute temporal resolution. If throughput is not a consideration, this design can enable in principle simultaneous monitoring of 14 cells (which are all imaged at once in one field of view, thus requiring no stage movement) at a 20-msec temporal resolution.

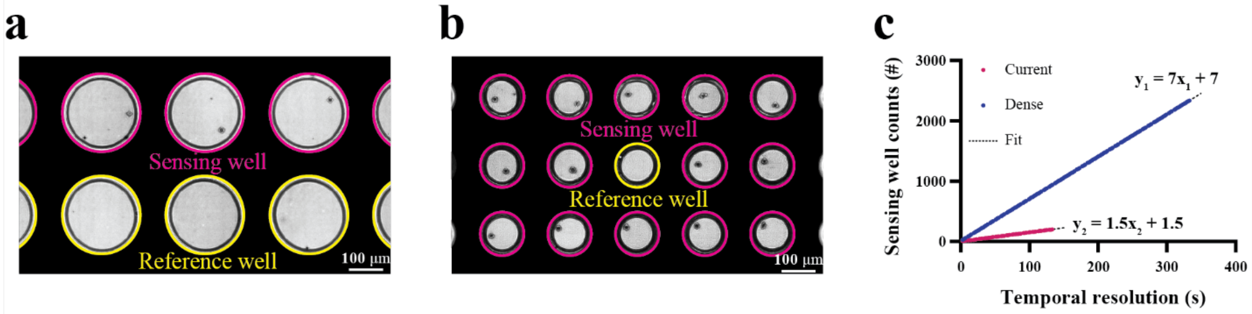

Supplementary Fig. 1 l Microwell array throughput and temporal resolution. **a**, an image of the field of view for the microwell array having 200 µm diameter wells and 50% reference occupancy. Half of the microwells are used as sensing wells and the other half for referencing. **b**, An image of the field of view for the dense microwell array having 100 µm diameter wells and 6.7% reference occupancy. The number of the sensing wells is increased from three to 14 while the number of reference wells is decreased from three to one. The magenta and yellow circles mark the sensing and reference wells, respectively. **c**, Plotting the number of sensing wells vs. the temporal resolution for the two designs shown in (**a**) and (**b**). The dots are the calculated values and the lines are the linear fittings.

Supplementary Discussion 2 | Deterministic single-cell seeding

An ultralow liquid dispensing technology was utilized to seed the single cells in the microwells (Supplementary Fig. 2a). It combines high accuracy single-cell isolation and picoliter reagent dispensing. The technology is compatible with different cell types with a diameter ranging from 0.5 to 80 µm. The minimum required sample volume is as low as one µL, which reduces the amount of consumed reagent by the process. Liquid dispensing is performed by a piezoelectric nozzle generating droplets of 200–600 pL volume depending on the nozzle parameters such as voltage and pulse. For single-cell isolation, the nozzle is coupled with an advanced image processing algorithm providing information on morphological parameters of the cells including elongation, circularity, and diameter (Supplementary Fig. 2b). As indicated in Supplementary Fig. 2c, the nozzle has two distinct zones, namely sedimentation and ejection (dashed red and green boxes) that are used for real-time image processing of the cell suspension. After defining the isolation parameters by the user, single cells are dispensed only if there is one desired cell in the green zone and the red zone is empty. By adjusting the cell suspension concentration, efficient deterministic single-cell isolation can be achieved (around 96 cells in less than three minutes). Supplementary Fig. 2d shows the schematic illustration of the plasmonic substrate and the open-top microwells formed by attaching the PDMS micromesh to the surface of the chip. To spot the single cells, first, the microwells were prefilled with cell culture medium supplemented with 1% v/v glycerol that prevents liquid evaporation. Then the nozzle spotted the single cells into the microwells. Supplementary Fig. 2e,f present the single-cell microwell array before and after seeding the cells with high precision. Humidity, temperature, and dew-point control along with ultra-gentle cell isolation prevent unwanted perturbation to the cells and guarantee their viability as indicated by the viability assay result in Supplementary Fig. 2g. After cell seeding, the cell culture medium was added to the customized chamber (Supplementary Fig. 2h), and consequently, the chamber was transferred to the microspore for secretion monitoring.

Our plasmonic substrates are in principle compatible with both random cell loading using manual pipetting and deterministic cell loading using a dispenser. In this study, we favored the piezoelectric liquid dispenser, which comes with certain advantages and disadvantages. For example, when the aim is to visualize the spatial distribution of the secreted materials from individual cells in the extracellular space over the surface, it is important to consider a “clear” area around each cell, enabling single-cell spatial secretion analysis independent of neighboring cells. In such cases, the piezoelectric dispenser not only guarantees single cells in the microwells, but it also optimizes the required substrate size by reducing the unused space. A smaller area is advantageous to reduce the chip cost and increase the temporal resolution (by minimizing the number of stage movement steps) for high-throughput microwell array applications. On the other hand, the piezoelectric liquid dispenser takes a longer time for cell seeding than the random approach, which can impose practical limitations for very large microwell arrays (> 10,000 cells).

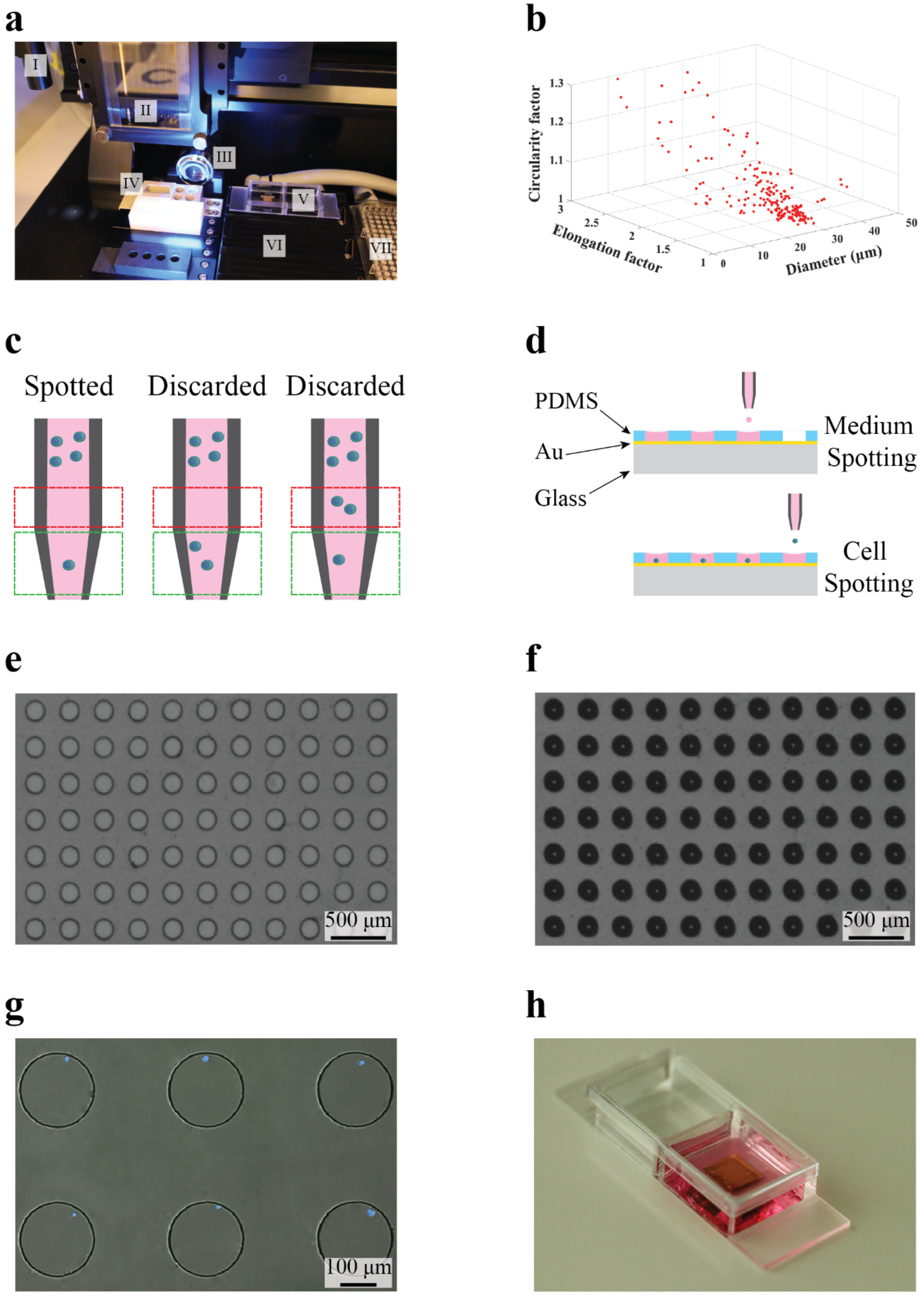

Supplementary Fig. 2 | Deterministic single-cell compartmentalization. **a**, Different elements of the spotting machine: (I) Head camera used for scanning the surface and positioning the nozzle, (II) Piezoelectric non-contact nozzle, (III) Droplet camera used for controlling the droplet size, distance from the nozzle, and stability, (IV) Wash solution reservoir, (V) Plasmonic single-cell microwell array in a customized chamber, (VI) Temperature controlled target holder, (VII) 384 well plate containing cell culture media and cell suspension. **b**, Three-dimensional scatter plot showing the extracted morphological parameters by the dispenser used to isolate the desired cells. **c**, Schematic illustration of the image processing protocol for single-cell spotting by the nozzle. The red and green dashed boxes represent the sedimentation and ejection zones, respectively. Single cells are spotted into the target wells when the sedimentation zone is empty and

there is only one cell in the ejection area. **d**, Schematic of the plasmonic single-cell microwell array. First, the medium is dispensed into the wells, and then the nozzle spots the cells. **e** and **f**, Images of the plasmonic microwell array before and after cell loading. **g**, Fluorescence images of the cells spotted into the microwells made of PDMS. The viable cells show blue signal and turn to green upon apoptosis. **h**, the Customized chamber in which the plasmonic substrate is immersed in cell culture medium.

a

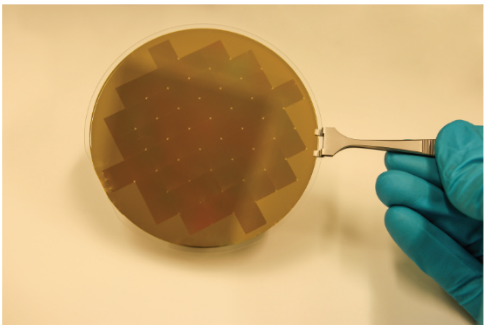

b

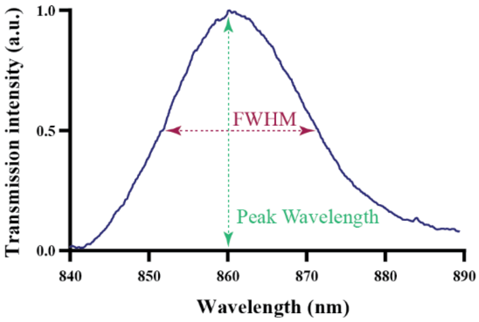

c

	Average value	Standard deviation
**Peak Wavelength [nm]**	860.7	2.1
**FWHM [nm]**	22.6	0.8

Supplementary Fig. 3 | Optical characterization of the plasmonic substrates. **a**, An image of the plasmonic nanohole array substrates fabricated in the wafer-scale. **b**, A typical transmission intensity spectrum for a plasmonic substrate measured in water indicating the peak wavelength and FWHM. **c**, Statistical evaluation of 98 plasmonic substrates from two wafers for their EOT peak position and FWHM.

Supplementary Discussion 3 | Functionalization validation with conventional SPR

To ensure the performance of the surface chemistry, we validated the functionalization steps with a Bionavis Ltd (MP-SPR Navi 210A VASA) SPR system. After the formation of the SAM layer on the SPR chip, we performed all the other steps for the functionalization in a real-time measurement to monitor the signal changes due to the binding interactions on the surface. As shown in Supplementary Fig. 4, after activating the surface with EDC/NHS, protein A/G was immobilized on the surface. The unreacted COOHs were deactivated using ethanolamine solution, and the excess protein-binding sites were blocked by the BSA solution. Finally, the performance of the surface for capturing the analyte was evaluated by injecting mouse IgGs, confirming the successful binding of IgG to the surface.

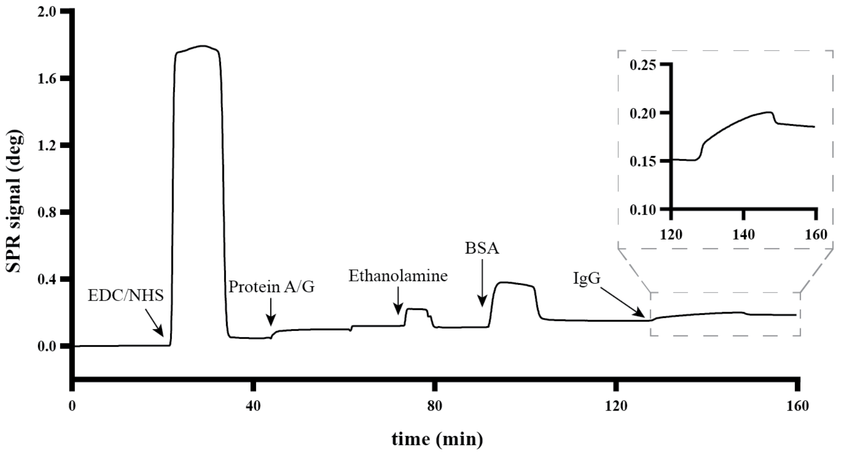

Supplementary Fig. 4 | Functionalization validation with conventional SPR. All the injections were performed at concentrations similar to those used to functionalize the gold nanohole array substrates. The IgG concentration was 10 ng/mL (shown by the inset).

Supplementary Discussion 4 | Functionalization uniformity

Since uniformity of the immobilized protein A/G is critical for the spatiotemporal mapping of the secretions, we evaluated the functionalization uniformity over the surface of the gold nanohole array plasmonic substrates. We assembled the functionalized substrate with a custom-made PDMS microfluidic unit consisting of microchannels of 500 µm width and 180 µm height and used a syringe pump for the sample injection. We injected a high concentration of mouse IgG (10 μg/mL) to completely occupy the binding sites on the surface and then monitored the intensity change distribution throughout the plasmonic substrate. Three different locations on the surface for two plasmonic substrates were investigated. Supplementary Fig. 5 shows the optical and processed images of a representative area before and after injection. It can be seen that before binding interactions (Supplementary Fig. 5d), little or no intensity variation was observed for all the pixels. In a similar way, after binding IgG molecules to the surface (Supplementary Fig. 5e), 11562 ± 420 a.u. intensity change was obtained for each pixel, illustrating the uniform distribution of the protein A/G on the surface.

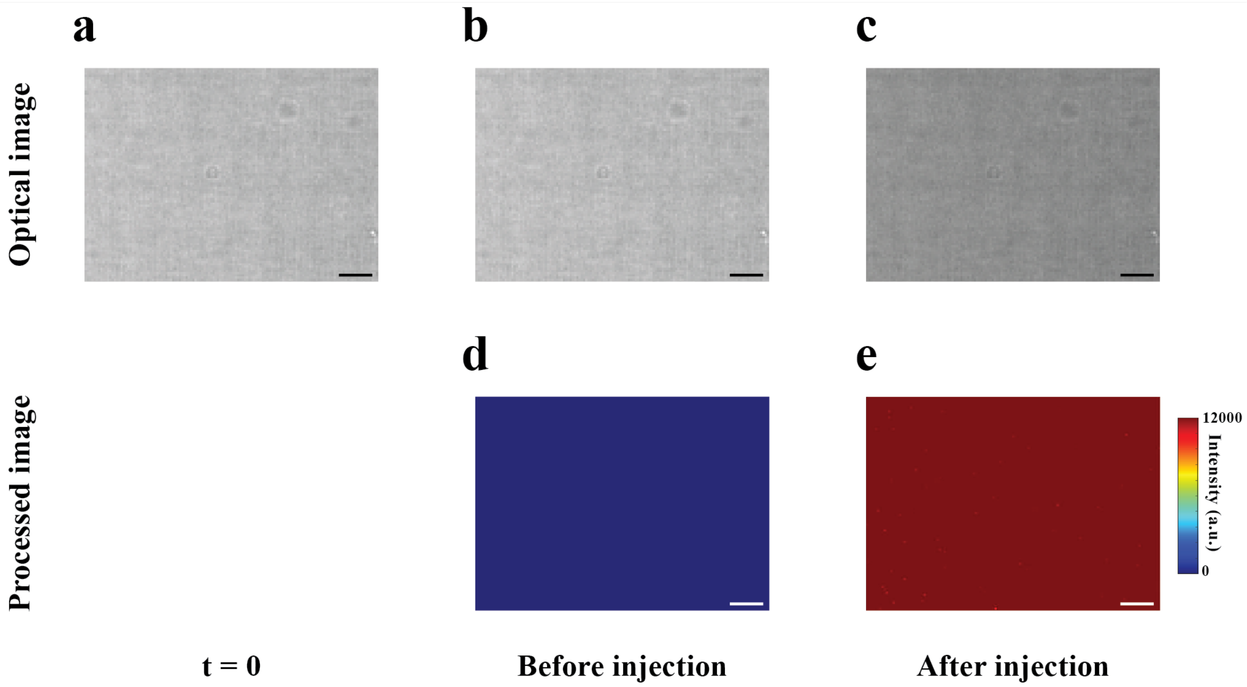

Supplementary Fig. 5 | Functionalization uniformity. **a**-**c**, Optical images of a representative area on the surface of the functionalized plasmonic substrate. **d** and **e**, Processed images of the surface before and after injecting IgG solution. The processed images were the result of pixel-wise subtraction between the first image (t=0) and the subsequent ones. The scale bars indicate 25 µm.

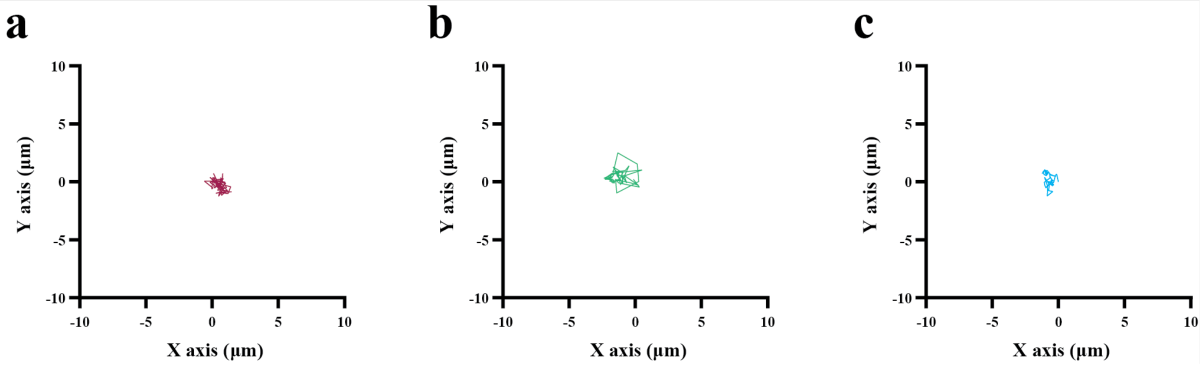

Supplementary Fig. 6 | Migration trajectory. **a**-**c**, Migration trajectories for the three representative hybridoma cells shown in Fig. 2c-e, respectively. The location of the cells at the onset of secretion was placed on the origins of the X and Y axes.

Supplementary Discussion 5 I Control experiments for specificity evaluation

We evaluated the specificity of the sensor by monitoring human lymphoblast K562 cells. Since these cells secrete a wide range of proteins but not IgGs, they are a suitable candidate to see whether the surface specifically captures IgGs or other biomolecules can also bind to the surface in a non-specific manner. We first evaluated the amount of proteins secreted by the K562 cells using a LEGENDplex kit. Supplementary Fig. 7a shows that this cell line produced a considerable amount of VEGF and a small amount of other proteins such as IL-6 and MCP-1 (Supplementary Fig. 7b). After ensuring the release of different biomolecules by the cells, we tested this cell line on our platform functionalized with protein A/G followed by a BSA blocking step. In order to demonstrate the necessity of capturing receptors on the sensor surface for specific detection of the secreted products and the effectiveness of the blocking process, we performed an experiment with the hybridoma cells on a PEG functionalized surface followed a by BSA blocking, in the absence of protein A/G.

Supplementary Fig. 7c,d compare the maximum TIC and secretion area values for these controls with the values for the hybridoma cells in Fig. 3. The results illustrate a significant difference in the signal levels. Especially, the average Max TICs, associated with the amount of secreted products, for these two controls are almost two orders of magnitude smaller than that of hybridomas. Although our current surface functionalization protocol is sufficient to have specific analyte binding and suppress non-specific adsorption for the employed cells, there could be scenarios for additional control (e.g., for target analytes secreted at a significantly lower amount than the other secreted molecules). In such cases, an in-line washing step (e.g. with microfluidics) can be considered to remove non-specific molecule adsorption and further improve specificity.

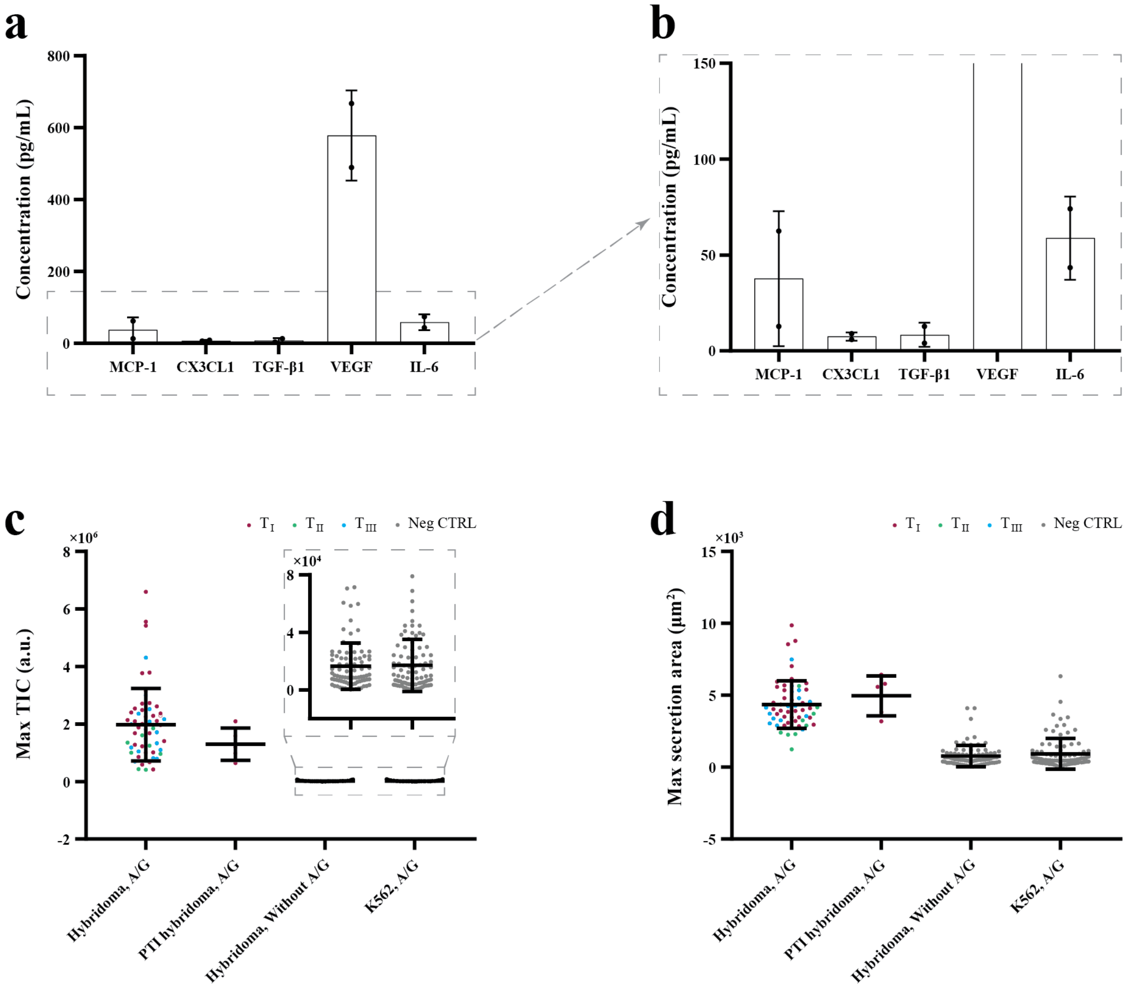

Supplementary Fig. 7 I Control experiments for specificity evaluation. **a** and **b**, Characterization of the secreted products by the K562 cells using a LEGENDplex™ kit. (**b**) shows the result for the proteins with a lower level of secretion compared with VEGF. Error bars represent the standard deviation between two replicates for each column. **c** and **d**, One-dimensional scatter plots that compare the maximum TIC and secretion area for all four experiments including hybridoma on the surface with protein A/G, PTI hybridoma

on the surface with protein A/G, hybridoma on the surface without protein A/G, and K562 on the surface with protein A/G. Inset in (**c**) shows the smaller changes for the control groups. The horizontal lines and error bars represent the mean value and standard deviation for each group. n = 160 for the hybridoma group, n = 168 for the PTI hybridoma group, n = 86 for the hybridoma without protein A/G functionalization group, and n = 93 for the K562 group; n is independent cells per experiment.

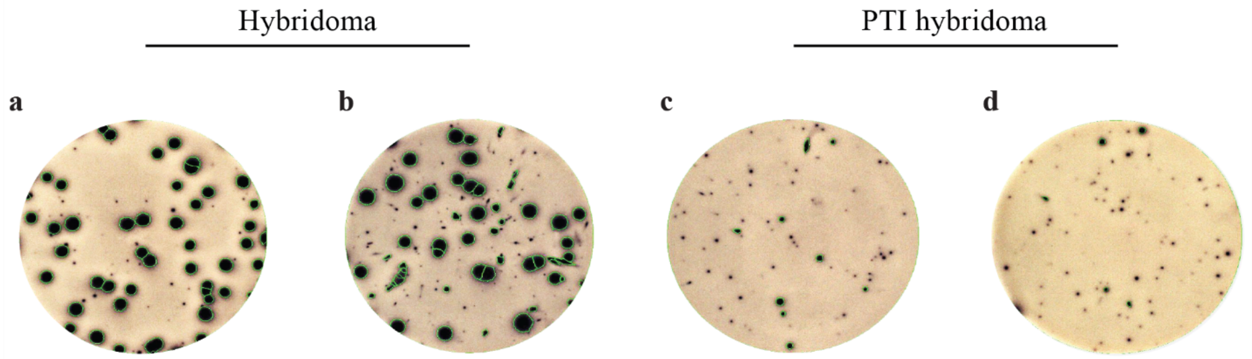

Supplementary Fig. 8 | ELISpot assay for mouse IgG detection in hybridoma cells. **a** and **b**, Two repetitions of IgG detection in untreated hybridoma cells using ELISpot. A population of 100 cells was incubated in each well for 12 hours. 48 cells in (**a**) and 53 cells in (**b**) were found secreting. **c** and **d**, IgG detection in PTI hybridomas as a control experiment. 9 cells in (**c**) and 5 cells in (**d**) were found secreting after 12 hours of incubation.

Supplementary Table 1 | Statistical analysis of hybridomas and PTI hybridomas.

Figure	Sample	Type	Mean	SD	Unit
3.g	Hybridoma	T_I, T_{II}, T_{III}	1978174	1263482	a.u.
	PTI hybridoma	T_I	1298400	559979	
3.h	Hybridoma	T_I, T_{II}, T_{III}	4346	1655	μm^2
	PTI hybridoma	T_I	4963	1388	
3.j	Hybridoma	T_I	303293	170668	a.u./h
		T_{II}	210888	87945	
		T_{III}	195388	109807	
		T_{III}	350246	212427	
	PTI hybridoma	T_I	511642	226066	
3.k	Hybridoma	T_I	1223	739.1	μm^2/h
		T_{II}	904.4	546.4	
		T_{III}	941.7	341.9	
		T_{III}	832.7	714.6	
	PTI hybridoma	T_I	2380	273.5	

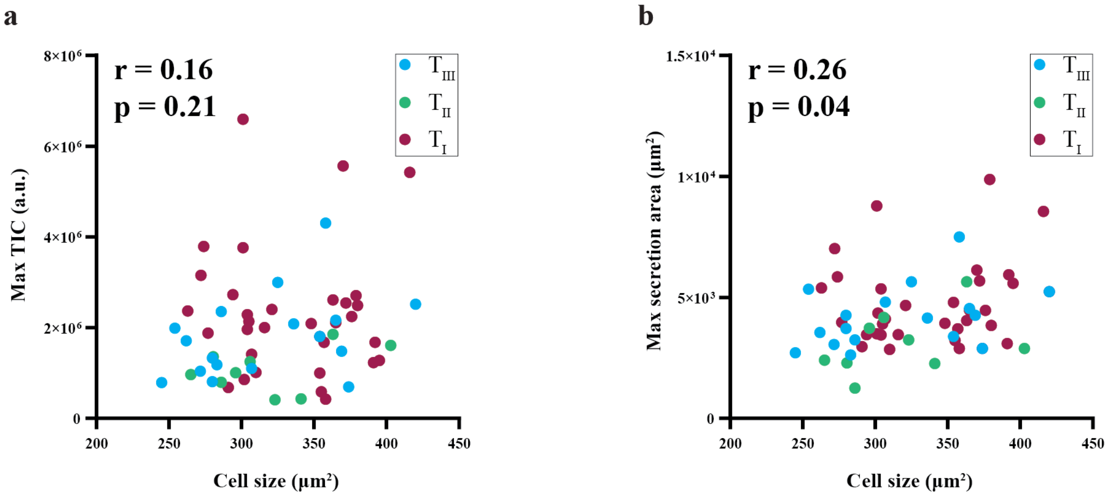

Supplementary Fig. 9 | Two-sided spearman's correlation analysis (data distribution is not normal). **a** and **b**, correlation analysis between the cell size (at the onset of secretion) and maximum TIC and maximum secretion area values for the untreated hybridoma cells, respectively. Correlation coefficients and P values are shown by r and p.

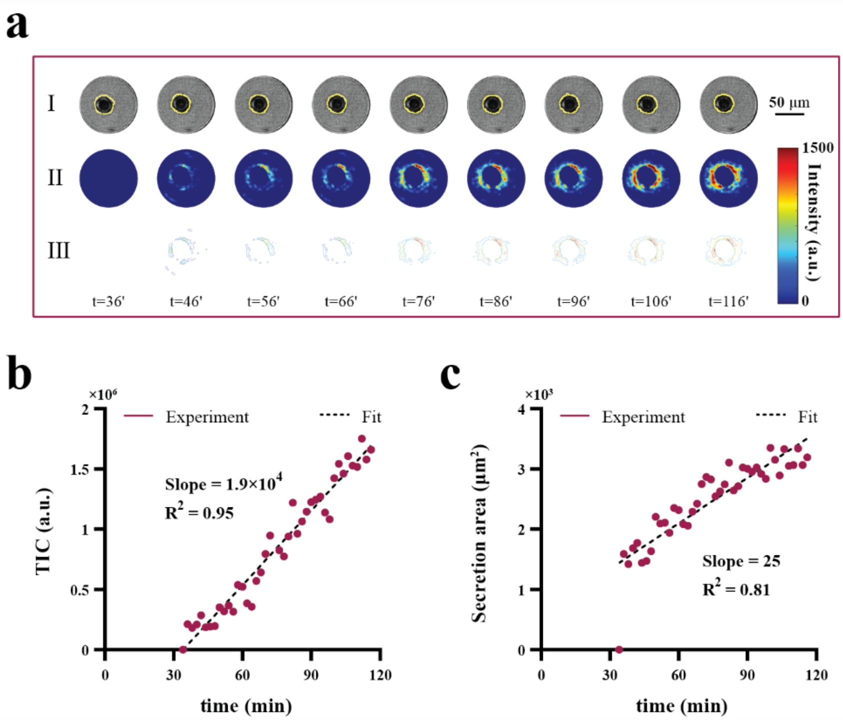

Supplementary Fig. 10 | Secretion monitoring with the dense plasmonic single-cell microwell array design having 100 μm diameter microwells with 6.7% reference occupancy. **a**, 4-D spatiotemporal secretion map for a representative secreting hybridoma cell. (I) are the time-lapse optical images, (II) are the spatiotemporal secretion maps, (III) are the secretion contour plots. **b** and **c**, TIC and secretion area curves indicating the amount of IgGs secreted over time and the area covered by the secretion on the sensor surface around the cell, respectively. Linear curve fitting is used to extract the secretion and adsorption rates (the slopes).

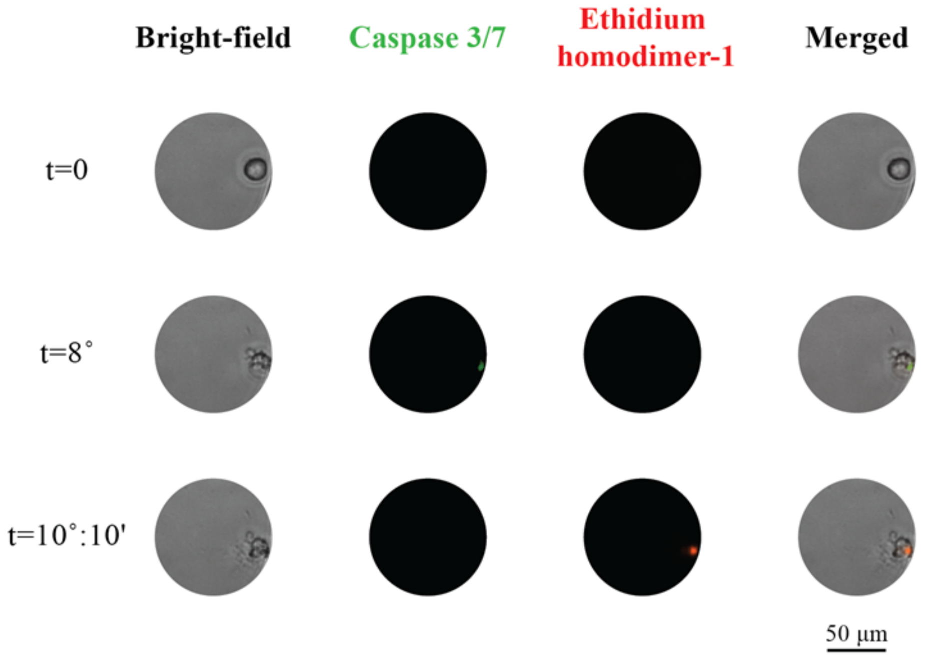

Supplementary Fig. 11 | Fluorescence imaging of a representative apoptotic cell. The cell showed membrane blebbing and caspase 3/7 activation (green fluorescence) followed by emitting red fluorescence resulting from binding of ethidium homodimer-1 to DNA when the membrane was compromised. The experiment was repeated twice with similar results.

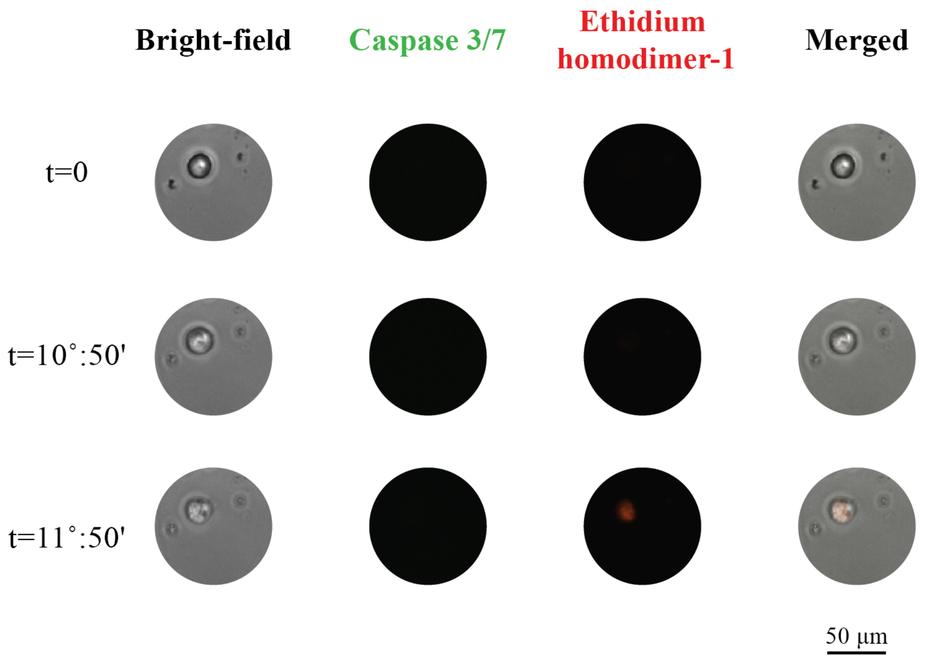

Supplementary Fig. 12 l Fluorescence imaging of a representative necroptotic cell. The cell experienced an increase in volume and only emitted red fluorescence resulting from DNA staining with ethidium homodimer-1 after membrane permeabilization without caspase 3/7 pathway activation. The experiment was repeated twice with similar results.

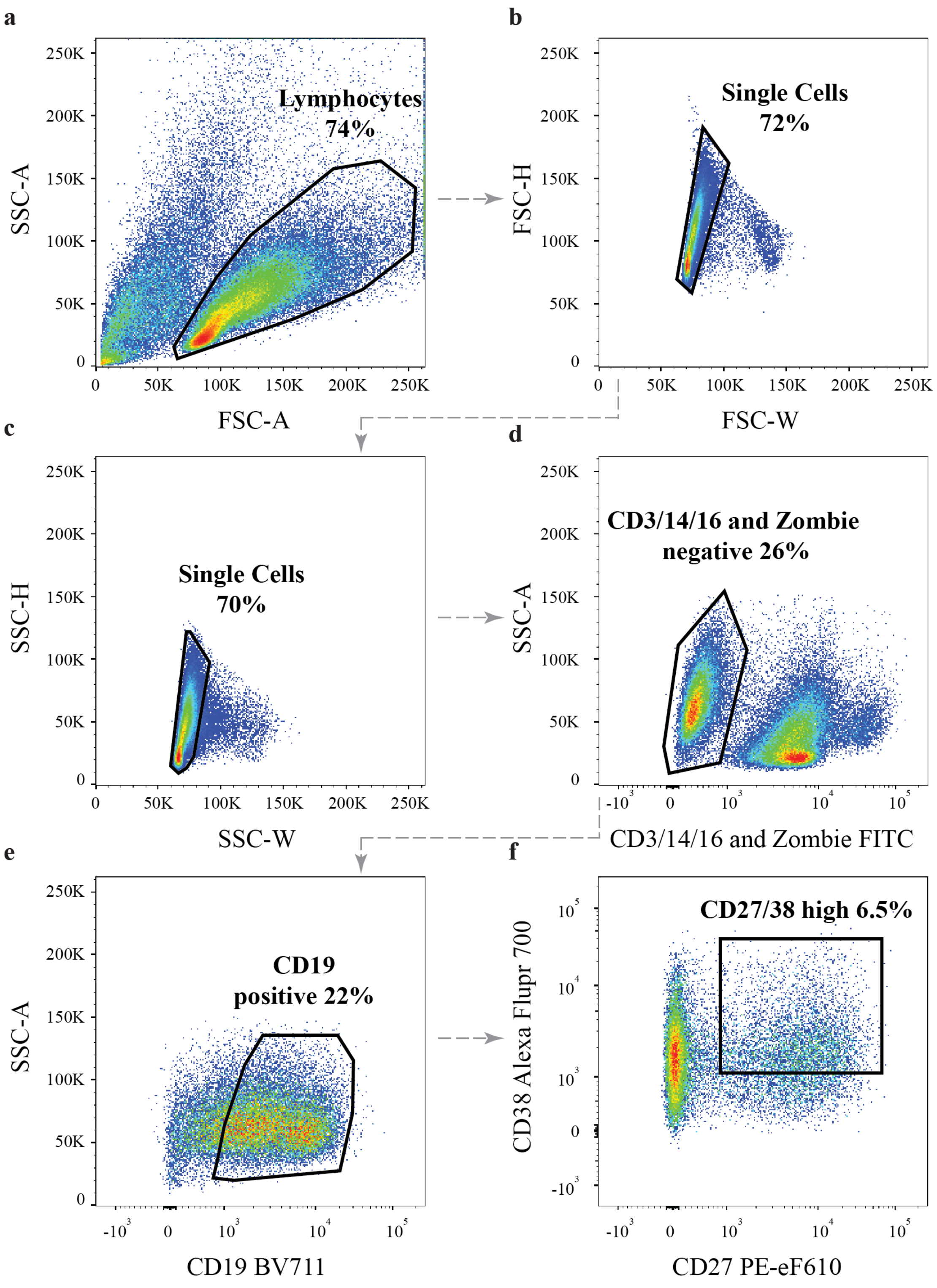

Supplementary Fig. 13 l Flow cytometric analysis of human PBMCs**. a**, Excluding cell debris and selecting lymphocytes in the population based on forward and side scatters. **b** and **c**, Excluding doublets and selecting singlets based on forward and side scatters, respectively. **d**, Live cells (Zombie negative) were gated on CD3/14/16 (negative) to exclude the monocytes, T, and natural killer cells. **e**, Gating the obtained population on CD19 (positive) to reach the B cells. **f**, Within the B cells, antibody-secreting cells were identified as CD27/38 (high) cells. Arrows show the gating strategy for detecting the potentially antibody-secreting cells.

Supplementary Table 2 | Statistical analysis of human PBMCs.

Figure	Sample	Type	Mean	SD	Unit
5.i	ASC	T_I, T_{II}, T_{III}	1085727	742388	a.u.
5.j	ASC	T_I, T_{II}, T_{III}	2788	1967	μm^2
5.k	ASC	T_I	421415	285482	a.u./h
		T_{II}	407755	305369	
		T_{III}	298050	-	
		T_{III}	346679	-	
5.l	ASC	T_I	1866	415.7	μm^2/h
		T_{II}	1318	539.5	
		T_{III}	3259.3	-	
		T_{III}	1170.1	-	

Supplementary Discussion 6 | Spatiotemporal analysis of IL-2 secretion from mouse EL-4 cells

In order to demonstrate that the platform is capable of detecting a wide range of biomolecules including those with small molecular weights (<20 kDa) and low secretion rates (<100 molecules/sec)[1], we evaluated mouse EL-4 cells from the T lymphoblast family to detect IL-2, a 15 kDa cytokine responsible for homeostasis and growth of T lymphocytes[2]. We first characterized the cells with a LEGENDplex kit to assess a panel of secreted cytokines and ensure IL-2 secretion upon proper stimulation with PMA and ionomycin[3] (see Methods for details). As shown in Extended Data Fig. 4a the stimulated cells produced a considerable amount of IL-2 compared with the control group. Besides IL-2, Extended Data Fig. 4b indicates that other cytokines were also produced. To specifically monitor IL-2 secretion, we functionalized the sensor based on streptavidin-biotin chemistry and immobilized anti-IL-2 antibody on the surface (see Methods for details). After loading the plasmonic single-cell microwell array with the individual EL-4 cells, we introduced the stimulation and monitored the single cells for 15 hours. Extended Data Fig. 4c shows a representative 4-D spatiotemporal secretion analysis for one of the secreting cells at selected time points along with the optical images for cell morphology monitoring during secretion (Supplementary Videos 27 and 28). Secretion contour plots are shown in Extended Data Fig. 4c, which indicate an asymmetric secretion pattern for this cell. Extended Data Fig. 4d,e present the TIC and secretion area curves for the EL-4 cell, illustrating type I secretion. As expected, the secretion and adsorption rates (1.67×10^5 a.u./h and 206.4 μm^2/h, respectively) were observed to be less than the average responses seen for the type I of the IgG secreting hybridoma cells (see Supplementary Table 1 for details).

Supplementary references

1. Han, Q., Bradshaw, E. M., Nilsson, B., Hafler, D. A. & Love, J. C. Multidimensional analysis of the frequencies and rates of cytokine secretion from single cells by quantitative microengraving. *Lab Chip* **10**, 1391–1400 (2010).
2. Boyman, O. & Sprent, J. The role of interleukin-2 during homeostasis and activation of the immune system. *Nat. Rev. Immunol.* **12**, 180–190 (2012).
3. Chatila, T., Silverman, L., Miller, R. & Geha, R. Mechanisms of T cell activation by the calcium ionophore ionomycin. *J. Immunol.* **143**, (1989).
