# Supplementary material for: High-throughput spatiotemporal monitoring of single-cell secretions via plasmonic microwell arrays
Source: Nat Biomed Eng. 2023 Apr 3;7(7):943–58. doi: 10.1038/s41551-023-01017-1 (PMC10365996; doi:10.1038/s41551-023-01017-1)
Supplement: Supplementary file 2 — Reporting Summary [file 41551_2023_1017_MOESM2_ESM.pdf]

nature portfolio

Corresponding author(s): Hatice Altug

Last updated by author(s): Jan 23, 2023

Reporting Summary

Nature Portfolio wishes to improve the reproducibility of the work that we publish. This form provides structure for consistency and transparency in reporting. For further information on Nature Portfolio policies, see our Editorial Policies and the Editorial Policy Checklist.

Statistics

For all statistical analyses, confirm that the following items are present in the figure legend, table legend, main text, or Methods section.

n/a	Confirmed	
☐	☒	The exact sample size (n) for each experimental group/condition, given as a discrete number and unit of measurement
☐	☒	A statement on whether measurements were taken from distinct samples or whether the same sample was measured repeatedly
☐	☒	The statistical test(s) used AND whether they are one- or two-sided<br>*Only common tests should be described solely by name; describe more complex techniques in the Methods section.*
☒	☐	A description of all covariates tested
☒	☐	A description of any assumptions or corrections, such as tests of normality and adjustment for multiple comparisons
☐	☒	A full description of the statistical parameters including central tendency (e.g. means) or other basic estimates (e.g. regression coefficient) AND variation (e.g. standard deviation) or associated estimates of uncertainty (e.g. confidence intervals)
☐	☒	For null hypothesis testing, the test statistic (e.g. F, t, r) with confidence intervals, effect sizes, degrees of freedom and P value noted<br>*Give P values as exact values whenever suitable.*
☒	☐	For Bayesian analysis, information on the choice of priors and Markov chain Monte Carlo settings
☒	☐	For hierarchical and complex designs, identification of the appropriate level for tests and full reporting of outcomes
☐	☒	Estimates of effect sizes (e.g. Cohen's d, Pearson's r), indicating how they were calculated

Our web collection on statistics for biologists contains articles on many of the points above.

Software and code

Policy information about availability of computer code

Data collection	NIS-Elements Advanced Research (Nikon's software version 5.01).
Data analysis	Custom-written Matlab script (version R2019), open-source ilastik (version 1.3.2), GraphPad Prism (version 9.2), Microsoft Excel 2019, FlowJo (version 10.8.0), and ImageJ (version 1.53f51). The data analysis for cytokine quantification using the LEGENDplex kits was conducted via LEGENDplex software (version 8.0).

For manuscripts utilizing custom algorithms or software that are central to the research but not yet described in published literature, software must be made available to editors and reviewers. We strongly encourage code deposition in a community repository (e.g. GitHub). See the Nature Portfolio guidelines for submitting code & software for further information.

Data

Policy information about availability of data

All manuscripts must include a data availability statement. This statement should provide the following information, where applicable:

- Accession codes, unique identifiers, or web links for publicly available datasets
- A description of any restrictions on data availability
- For clinical datasets or third party data, please ensure that the statement adheres to our policy

The main data supporting the results in this study are available within the paper and its Supplementary Information. The raw and analysed datasets generated during the study are too large to be publicly shared, yet they are available for research purposes from the corresponding author on reasonable request.

Field-specific reporting

Please select the one below that is the best fit for your research. If you are not sure, read the appropriate sections before making your selection.

☒ Life sciences ☐ Behavioural & social sciences ☐ Ecological, evolutionary & environmental sciences

For a reference copy of the document with all sections, see nature.com/documents/nr-reporting-summary-flat.pdf

Life sciences study design

All studies must disclose on these points even when the disclosure is negative.

Sample size

For each experiment, either with cell lines or human peripheral blood mononuclear cells (PBMCs), the exact number of cells is indicated in the paper. Because a goal of this study was to increase the throughput from a few tens of cells to hundreds of cells, we used the full potential of the system to have the maximum number of cells. No sample-size calculation was performed.

Data exclusions

No data were excluded.

Replication

The experiments for cytokine quantification and ELISpot assays were performed in duplicate, and the results were reproducible. Data of all replicates are included in the paper. The experiments with hybridoma cells for antibody detection were performed in duplicate, with reproducible results (yet only the result for one of the experiments is shown).

Randomization

No randomization was used because the study was focused on demonstrating the sensor performance rather than evaluating and comparing clinical samples.

Blinding

No blinding was performed.

Reporting for specific materials, systems and methods

We require information from authors about some types of materials, experimental systems and methods used in many studies. Here, indicate whether each material, system or method listed is relevant to your study. If you are not sure if a list item applies to your research, read the appropriate section before selecting a response.

Materials & experimental systems

n/a	Involved in the study	
☐	☒	Antibodies
☐	☒	Eukaryotic cell lines
☒	☐	Palaeontology and archaeology
☒	☐	Animals and other organisms
☐	☒	Human research participants
☒	☐	Clinical data
☒	☐	Dual use research of concern

Methods

n/a	Involved in the study	
☒	☐	ChIP-seq
☐	☒	Flow cytometry
☒	☐	MRI-based neuroimaging

Antibodies

Antibodies used

Pierce recombinant protein A/G (Cat# 21186, 50µg/mL), AlexaFluor700 anti-human CD38 (Cat# 56-0389-42, 1:50), and PE-eF610 anti-human CD27(Cat# 61-0279-42, 1:50) were obtained from Thermo Fisher Scientific. FITC anti-human CD16 (Cat# 302005, 1:200), FITC anti-human CD14 (Cat# 301803, 3:500), FITC anti-human CD3 (Cat# 300405, 1:100), and Brillant Violet 711 anti-human CD19 (Cat# 302245, 1:200) were purchased from Biolegend. Biotinylated mouse IL-2 antibody (Cat# 3441-6-250, 50µg/mL), anti-human IgG (Cat# 3850-3-250, 15 µg/mL), biotinylated anti-human IgG (Cat# 3850-6-250, 1µg/mL), anti mouse IgG (kit Cat# 3825-2A, 15 µg/mL), and Biotinylated anti-mouse IgG (kit Cat# 3825-2A, 1µg/mL) were purchased from Mabtech.

Validation

All antibodies were validated by the manufacturers and used according to the manufacturers' protocols. For experiments with the single-cell microwell array, SPR characterization was performed (Supplementary information).

Eukaryotic cell lines

Policy information about cell lines

Cell line source(s)

Clonal mouse hybridoma cells (anti-CD45.1) and mouse EL-4 cells (TIB-39) were kindly provided by Dr. Anne Wilson from the University of Lausanne. K562 cells (CCL-243) were obtained from ATCC.

Authentication

In addition to the flow cytometry analyses done by Dr. Wilson, ELISpot assays were used to check the secretory behaviours of the cells.

Mycoplasma contamination	The cell lines tested negative for mycoplasma contamination.
Commonly misidentified lines (See ICLAC register)	No commonly misidentified cells lines were used.

Human research participants

Policy information about studies involving human research participants

Population characteristics	PBMCs derived from anonymised buffy coats obtained from healthy blood donors from the transfusion center at the University hospitals of Geneva who met the local criteria for blood donation. No additional information on population characteristics was available.
Recruitment	Samples were obtained from the local donor blood bank, for which participants volunteered, and the selection of the Buffy coat was random.
Ethics oversight	The Swiss Transfusion Center of Geneva, Switzerland.

Note that full information on the approval of the study protocol must also be provided in the manuscript.

Flow Cytometry

Plots

Confirm that:

☒ The axis labels state the marker and fluorochrome used (e.g. CD4-FITC).

☒ The axis scales are clearly visible. Include numbers along axes only for bottom left plot of group (a 'group' is an analysis of identical markers).

☒ All plots are contour plots with outliers or pseudocolor plots.

☒ A numerical value for number of cells or percentage (with statistics) is provided.

Methodology

Sample preparation	Sample preparations for flow-cytometry analysis are detailed in Methods.
Instrument	FACSAria II instrument (BD Biosciences)
Software	FlowJo (version 10.8.0)
Cell population abundance	The abundance of antibody-secreting cells was 6.5% and 2.3% in the two donor samples used in this study.
Gating strategy	First, we excluded cell debris and selected lymphocytes in the population based on forward and side scatters. Then, we excluded doublets and selected singlets based on forward and side scatters, respectively. Live cells (Zombie negative) were gated on CD3/14/16 (negative) to exclude the monocytes, T cells and natural killer cells. Next, we gated the obtained population on CD19 (positive) to reach the B cells. Within the B cells, antibody-secreting cells were identified as CD27/38 (high) cells.

☒ Tick this box to confirm that a figure exemplifying the gating strategy is provided in the Supplementary Information.
